# Transcriptomic and Metabolic Insight Into Flavonoid Biosynthesis Underlying Black and Yellow Seed Coat Color Variation in Soybean ( *Glycine max* )

**DOI:** 10.1002/pld3.70153

**Published:** 2026-02-20

**Authors:** Kahee Moon, Prakash Basnet, Seung Young Choi, Beom‐Soon Choi, Grace Lachica, Nam‐Il Park, Kyong‐Cheul Park, Taeyoung Um, Ik‐Young Choi

**Affiliations:** ^1^ Department of Agriculture and Life Industry Kangwon National University Chuncheon Republic of Korea; ^2^ NBIT Co., Ltd. Chuncheon Republic of Korea; ^3^ Department of Plant Science Gangneung‐Wonju National University Gangneung Republic of Korea; ^4^ Department of Smart farm and Agricultural Industry Kangwon National University Chuncheon Republic of Korea

**Keywords:** anthocyanin, flavonoid biosynthesis, *Glycine max*, proanthocyanidin, seed coat color, transcriptome

## Abstract

Soybean ( *Glycine max* ) seed coat color variation is determined by the accumulation of flavonoid‐derived pigments, although the molecular mechanisms underlying this trait remain poorly understood. This study integrated RNA sequencing (RNA‐Seq) and high‐performance liquid chromatography (HPLC)–based metabolite measurements to investigate black and yellow seed coat soybean lines derived from the same genetic background. Metabolite analysis revealed significantly higher total phenolic content (TPC), total flavonoid content (TFC), total anthocyanin content (TAC), total proanthocyanidin content (TPAC), and antioxidant activity (DPPH, ABTS) in black seed coats, whereas yellow seed coats exhibited elevated total isoflavone content (TIC). RNA‐seq at 110 days after sowing (DAS) identified differential expression of flavonoid pathway genes associated with these metabolic differences. Genes upregulated in black seed coats included flavanone 3‐hydroxylase (*F3H*), anthocyanidin synthase (*ANS*), UDP‐glycosyltransferases (*UGT78D2*, *UGT79B6*), and glutathione *S*‐transferase (*GSTF11*), encoding enzymes reported to function in anthocyanin biosynthesis, glycosylation, and vacuolar transport, respectively. Conversely, leucoanthocyanidin reductase (*LAR*) genes showed higher expression in yellow seed coats despite lower proanthocyanidin (PA) levels, whereas *LAC5* exhibited black seed–specific expression consistent with potential PA polymerization activity. R2R3‐MYB transcription factor genes along with small heat shock protein genes (*sHSPs*) were also upregulated in black seed coats, suggesting candidate regulatory roles in pigmentation and stress responses. Cytochrome P450 genes showed preferential expression in yellow seed coats, consistent with isoflavonoid pathway activation. Together, these findings elucidate the genetic and metabolic regulation of seed coat color in soybean and identify candidate genes relevant for functional breeding and genomics research.

## 1 Introduction

Soybean ( *Glycine max* [L.] Merrill) is widely recognized as one of the most significant crops due to its high economic value and broad consumption in agriculture and industry (Lee et al. 2015; Ncube et al. 2022). Currently, it ranks among the world's seven most important crops for its high protein (~40%), oil content (~20%), and richness in bioactive compounds (Medic et al. 2014; USDA 2024; FAOSTAT 2025). Soybean cultivation supports diverse industrial sectors, including food production, animal feed, edible oil, biodiesel, pharmaceuticals, and bioplastics (Choi et al. 2008; Waqas et al. 2015; Dey et al. 2023). Accordingly, ensuring high‐quality soybean production is essential, and seed coat color serves as a critical factor influencing quality traits.

The seed coat color is an important agronomic trait influencing seed quality and trait evolution (Qiu et al. 2021). Soybean seeds exhibit broad phenotypic diversity, with seed coat color being a key trait influencing both quality grading and commercial value (Hosamani et al. 2013; Liu et al. 2017). Seed coats range from monochromatic (black, brown, yellow, green) to patterned types (bicolor, mottled, spotted) (Voysest 2000; Song et al. 2016; C. Zhang, Zhang, et al. 2023). Whereas yellow seed coats dominate in commercial production, black seed coats are increasingly valued in functional food markets and specialty products (Kafer et al. 2023; Lee et al. 2023). The seed coat is critical for seed quality, modulating water uptake, disease resistance, and structural integrity (Moïse et al. 2005). Relative to yellow or green seeds, black and brown seeds typically exhibit stronger dormancy, better overwintering capacity, enhanced pathogen resistance, and improved seed longevity (Kyle and Dickson 1988; Campa et al. 2010; L. Zhang, Jia, et al. 2023). Black soybeans are also enriched in bioactive compounds such as anthocyanins, saponins, and proteins, which confer various health benefits, including anticancer, anti‐inflammatory, antidiabetic, anti‐obesity, nephroprotective, anti‐arthritis, and lipid‐lowering effects (Wu and Paice 2011; Chaturvedi et al. 2012; Ganesan and Xu 2017; Wu et al. 2017), all of which have nutritional and commercial significance.

Several genetic technologies, including QTL mapping and genome‐wide association studies (GWAS), have facilitated the identification of multiple genes and QTLs underlying seed coat color traits in soybean (Yang et al. 2023). Previous studies have identified several loci involved in seed coat pigmentation, including I (inhibitor), R (brown), T (tawny), O, and W1 (Yang et al. 2010). The I locus produces small interfering RNAs that inhibit chalcone synthase expression, resulting in yellow pigmentation (Todd and Vodkin 1993; Tuteja et al. 2004). Dominant alleles at the R and T loci enhance anthocyanin biosynthesis (Zabala and Vodkin 2014).

Advancements in transcriptomics have streamlined the elucidation of molecular mechanisms governing target traits in plants (Yu et al. 2025). Metabolomic profiling is increasingly recognized as a promising approach for advancing soybean crop improvement (Joshi and Xu 2022). Furthermore, integrating transcriptomics with metabolomics provides enhanced resolution of gene to metabolite networks and trait regulation (Hirai et al. 2004). Limited research has been done previously using the recombinant inbred lines (RILs). Dong et al. (2020) employed simple sequence repeat (SSR) markers to analyze seed coat color variation in an F_2_ population derived from the yellow‐seeded cultivar. Liu et al. (2021) analyzed the SojacsSLP5 wild soybean population, derived from NN1138‐2 × N24852, integrating SNP mapping with RNA‐Seq to identify two candidate genes for seed coat color. X. Wang et al. (2022) integrated metabolomic and transcriptomic analyses to elucidate the molecular mechanisms underlying testa pigmentation in peanut and Ma et al. (2023) in mung bean. Previous studies have identified key biosynthetic pathways and candidate genes involved in seed coat color; however, many lacked integrations of RILs representing segregating seed coat phenotypes and comprehensive metabolite profiling.

This study investigated the molecular mechanism underlying seed coat color variation in soybean through integrated transcriptomic (RNA‐seq) and major metabolite measurements (HPLC) approaches. RILs were developed from a black seed coat Korean landrace (KWS19, female parent) and a yellowish‐brown seed coat semi‐wild type (T191199, male parent). Two F9 lines with black and yellow seed coats were selected. Both progeny and parental lines were analyzed for phenolic, flavonoid, anthocyanin, proanthocyanidin (PA), and isoflavone contents, along with antioxidant activities. RNA‐seq of developing seeds identified differentially expressed genes (DEGs) associated with seed coat pigmentation. Functional enrichment analyses using Gene Ontology (GO) and Kyoto Encyclopedia of Genes and Genomes (KEGG) pathways analysis categorized the functions of these DEGs. Expression levels of key flavonoid biosynthesis genes were further validated by quantitative RT‐qPCR. These findings provide mechanistic insight into how transcriptional changes drive flavonoid metabolism and identify potential targets for improving seed quality traits in soybean breeding.

## 2 Materials and Methods

### 2.1 Plant Materials

The plant materials used in this study consisted of two selected soybean lines (i.e., 2023_437B and 2023_437Y). These lines were derived from RILs developed using the black seed coat Korean soybean variety KWS19_401 ( *G. max* ) as the maternal parent and a semi‐wild type with a brownish yellow seed coat, T191199_402 ( *G. max* × *Glycine soja* hybrid) used as the paternal parent. Both lines were cultivated via the SSD method, resulting in clearly distinguishable seed coat colors in the F9 generation. Cultivation was initiated on June 26, 2023, at the experimental field of Kangwon National University (37.9534° N, 127.7459° E), with all lines categorized as late‐maturing. Plants were grown for approximately 140 days.

### 2.2 Phenotype Observation and Sampling

To monitor seed coat color changes, seeds were sampled at 10‐day intervals from 100 to 130 DAS. At 110 DAS, when the seed coat color differences between the two lines were most prominent, seeds were harvested for RNA transcriptome analysis. For each seed coat color, three independent biological replicates were collected, each consisting of seeds pooled from different plants grown under the same conditions. Immediately after harvest, individual seeds were frozen in liquid nitrogen and stored at −80°C. Fully mature seeds from the same field plots were used for metabolite analysis.

### 2.3 Metabolite Analysis

#### 2.3.1 Isoflavone and PA Analysis by HPLC

Isoflavone content was determined in freeze‐dried seed powders extracted with 70% ethanol, with three independent replicates performed for each measurement. Briefly, 0.1 g of powdered sample was incubated with 1 mL of 70% ethanol at room temperature for 24 h, centrifuged, and the supernatant was filtered through a 0.22‐μm membrane prior to chromatographic analysis. Isoflavones (daidzin, glycitin, genistin, daidzein, glycitein, and genistein) were quantified by HPLC using a diode array UV‐Vis detector and a C18 column, with a water/acetonitrile mobile phase containing 0.1% formic acid under a linear gradient at 0.7 mL/min, 40°C, and detection at 280 nm. Calibration curves were generated from external standards (62.5–1000 μg/mL), and individual isoflavone concentrations in seed extracts were calculated from these standard curves.

PA content was determined by HPLC using a protocol modified from Yi et al. (2019). Freeze‐dried seed extracts were analyzed on the same HPLC system and C18 column described for isoflavones, with a mobile phase of water (0.1% formic acid, Solvent A) and acetonitrile (0.1% formic acid, Solvent B) at a flow rate of 0.7 mL/min, injection volume of 10 μL, and column temperature of 40°C. The gradient program was set to 20% B at 0 min, 28% B at 20 min, 60% B at 30 min, 80% B at 32 min, then returned to 20% B at 34 min, and held until 40 min, with detection at 280 nm. PA standards obtained from ChemFace (Wuhan, China) were used to generate calibration curves over the range 62.5–1000 μg/mL, and sample concentrations were calculated from these standard curves.

#### 2.3.2 Total Phenol Content (TPC) Analysis

Total phenolic content (TPC) was determined using a modified Folin–Ciocalteu colorimetric assay. Briefly, 70% ethanol extracts were adjusted to a concentration of 10,000 μg/mL, and 100 μL of each sample was mixed with 50 μL Folin–Ciocalteu reagent and incubated for 3 min. After addition of 300 μL 20% Na_2_CO_3_ and a further 15 min incubation, 1000 μL distilled water was added, the mixture was centrifuged, and 200 μL of the supernatant was transferred to a 96‐well plate for measurement at 738 nm using a microplate reader. TPC was expressed as mg gallic acid equivalents (GAE) per g dry weight, calculated from a gallic acid calibration curve.

#### 2.3.3 Total Flavonoid Content (TFC) Analysis

Total flavonoid content (TFC) was measured following a slightly modified method from NFRI (1990). Samples extracted with 70% ethanol were diluted to 100,000 μg/mL. A volume of 500 μL sample solution was mixed with 100 μL of 10% aluminum nitrate and 100 μL of 1 M potassium acetate and then incubated at room temperature for 40 min. The reaction mixture was transferred into a 96‐well plate, and absorbance was measured at 405 nm. A calibration curve using quercetin as the standard was established (y = 0.0066x + 0.0253, *R* ^2^ = 0.998).

#### 2.3.4 Total Anthocyanin Content (TAC) Analysis

Total anthocyanin content (TAC) was analyzed following the method described by Giusti and Wrolstad (2001). Samples (0.1 g) were mixed with 16 mL of 0.1% HCl in methanol and centrifuged, and 1 mL of the supernatant was collected. The supernatant (1 mL) was diluted with an additional 4 mL of 0.1% HCl in methanol. The diluted sample (1 mL) was divided into two aliquots for analysis. Aliquot A was mixed with 4 mL of 0.025 M potassium chloride buffer (pH 1.0), and aliquot A' was mixed with 4 mL of 0.4 M sodium acetate buffer (pH 4.5). Absorbance of both solutions was measured at 520 and 700 nm using a spectrophotometer. Distilled water was used as a reference. TAC (mg/L) was calculated using the pH differential method as

$$\begin{array}{c} \text{Total anthocyanins}\;\left(\mathrm{mg}/L\right)=\left[\left(A_{52\text{0nm}}-A_{70\text{0nm}}\right)\;\mathrm{pH}\;1.0-\left(A_{52\text{0nm}}-A_{70\text{0nm}}\right)\;\mathrm{pH}\;4.5\right]\times \mathrm{MW}\times \mathrm{DF}\times 1000/\left(\varepsilon \times l\right), \end{array}$$

where MW is the molecular weight of the reference anthocyanin, DF is the dilution factor, ε is the molar extinction coefficient, and l is the path length (1 cm).

#### 2.3.5 DPPH (2,2‐Diphenyl‐1‐Picrylhydrazyl) and ABTS 2,2′‐Azino‐Bis (3‐Ethylbenzothiazoline‐6‐Sulfonic Acid) Radical Scavenging Assay

Antioxidant activity was evaluated using DPPH and ABTS radical scavenging assays following modified methods of Blois (1958) and Re et al. (1999), respectively. For both assays, samples extracted with 70% ethanol were diluted to 1000 μg/mL. In the DPPH assay, 100 μL of diluted sample was mixed with 100 μL of 0.15 mM DPPH solution and incubated in the dark for 30 min at room temperature, with absorbance recorded at 519 nm. For the ABTS assay, the ABTS+ working solution was prepared by reacting 7.4 mM ABTS with 2.6 mM potassium persulfate for 24 h and diluting to an absorbance of 0.7 ± 0.03 at 738 nm. A total of 20 μL of the sample was combined with 180 μL of ABTS+ solution and incubated for 10 min prior to absorbance measurement at 738 nm. Ascorbic acid was used as the reference standard in both assays, and radical scavenging activity was calculated as electron‐donating ability (EDA, %). Radical scavenging activity was expressed as electron‐donating ability (EDA, %) and calculated as

$$\mathrm{EDA}\;\left(\%\right)=\left[1-\left(\text{absorbance of sample}/\text{absorbance of control}\right)\right]\times 100$$

### 2.4 RNA Extraction and Transcriptome Analysis

Total RNA was extracted using the GeneAll Ribospin Plant kit (GeneAll Biotechnology, Korea) using three independent biological replicates for each seed coat color (black and yellow). DNase I (Sigma, USA) treatment was done to remove genomic DNA. RNA concentration and purity were measured using a Multiskan Sky microplate spectrophotometer with SkanIt Software 6.0.2 (Thermo Fisher Scientific, USA). RNA‐seq libraries were prepared from 1 μg of total RNA via poly‐A selection, followed by cDNA synthesis, and sequenced on a DNBSEQ‐G400RS platform (MGI Tech Co. Ltd., Shenzhen, China) at Next Bio Information Technology (Chuncheon, Gangwon‐do, Korea) to generate 150‐bp paired‐end reads. The raw sequencing data were deposited into the NCBI SRA database with accession number GSE313134. Raw reads were quality‐trimmed and adapter sequences removed using Trimmomatic v0.39 (http://www.usadellab.org/cms/?page=trimmomatic), discarding reads shorter than 50 bp or with average quality < Q20. Clean reads were aligned to the *G. max* reference genome (Wm82.a4.v1) using HISAT2 v2.1.0 (http://daehwankimlab.github.io/hisat2/), and read counts per gene were obtained using HTSeq v0.11.2. The read counts for each gene were calculated using HTSeq V0.11.2 (https://htseq.readthedocs.io/en/master/). DEG analysis was performed using DESeq2 V1.38.0 (https://www.bioconductor.org/packages/2.10/bioc/html/DESeq.html) based on gene read counts. DEGs were identified with DESeq2 v1.38.0 (https://www.bioconductor.org/packages/release/bioc/html/DESeq2.html) using thresholds of |log2FC| ≥ 2, *p*‐value ≤ 0.05, and adjusted *p*‐value (*q*‐value) ≤ 0.05.

### 2.5 Gene Annotation and Functional Enrichment Analysis

Gene annotation was performed through sequential functional analyses. Protein‐coding sequences were first compared against the NCBI nr database using BLAST v2.12.0+ (ftp://ftp.ncbi.nlm.nih.gov/blast/executables/blast+/LATEST/) to identify homologous proteins. Protein domains and motifs were annotated using InterProScan v5.56–89.0 (https://www.ebi.ac.uk/interpro/search/sequence/), and GO terms were assigned with BLAST2GO v6.0.3 (https://www.blast2go.com/). Functional information was further validated using SoyBase (https://www.soybase.org/) and UniProt (https://www.uniprot.org/), whereas homologs in *Arabidopsis thaliana* were identified via TAIR (https://www.arabidopsis.org/) to improve annotation accuracy. GO classification was conducted for biological process, cellular component, and molecular function categories. The KEGG pathway analysis was performed via BLAST2GO (https://www.blast2go.com/). The enrichment analyses were performed using a significance threshold of *p* ≤ 0.05 to identify overrepresented biological pathways.

### 2.6 RT‐qPCR

Reverse‐transcription quantitative PCR (RT‐qPCR) analysis was performed using TB Green Premix Ex Taq II (TaKaRa, Japan) on a CronoS‐TAR Real‐Time PCR system (Clotech, Japan). cDNA was synthesized from 2 μg of total RNA using the Cycle‐Script reverse transcription protocol and kit. PCR reactions were initiated with a denaturation step at 95°C for 1 min, followed by 45 cycles of 95°C for 5 s, 60°C for 10 s, and 72°C for 10 s. Melting curve analysis was conducted by incrementally increasing the temperature from 72°C to 95°C in steps of 0.5°C to verify the specificity of amplification products, confirmed by the presence of a single fluorescence peak. *Actin11* (*Act11*) was used as an internal reference gene for normalization of gene expression levels. Primers were designed using NCBI Primer‐BLAST (Table S2), and all primers were synthesized by Bionicsro.co.kr. Quantitative PCR was conducted with three technical replicates for each sample. Relative gene expression levels were calculated using the 2^−ΔΔ^CT method.

### 2.7 Statistical Analysis

Statistical analysis was performed using IBM SPSS Statistics 26 software (IBM, Armonk, NY, USA). Pearson's correlation coefficients (*r*) were calculated to evaluate the relationships among isoflavone content, TPC, TFC, antioxidant activities (DPPH and ABTS), TAC and total proanthocyanidin content (TPAC), and LAR gene expression levels. All metabolite and gene expression measurements were obtained from three biological replicates per line, each analyzed with three technical replicates. The significance of correlation coefficients was evaluated using a two‐tailed Student's *t*‐test at *α* = 0.05 and *α* = 0.01, denoted as * (*p* < 0.05) and ** (*p* < 0.01), respectively. The strength of correlation coefficients was interpreted as weak (0.1–0.3), moderate (0.3–0.7), or strong (> 0.7).

## 3 Results

### 3.1 Development and Characterization of Black and Yellow Seed Coat Soybean Lines

**FIGURE 1:**
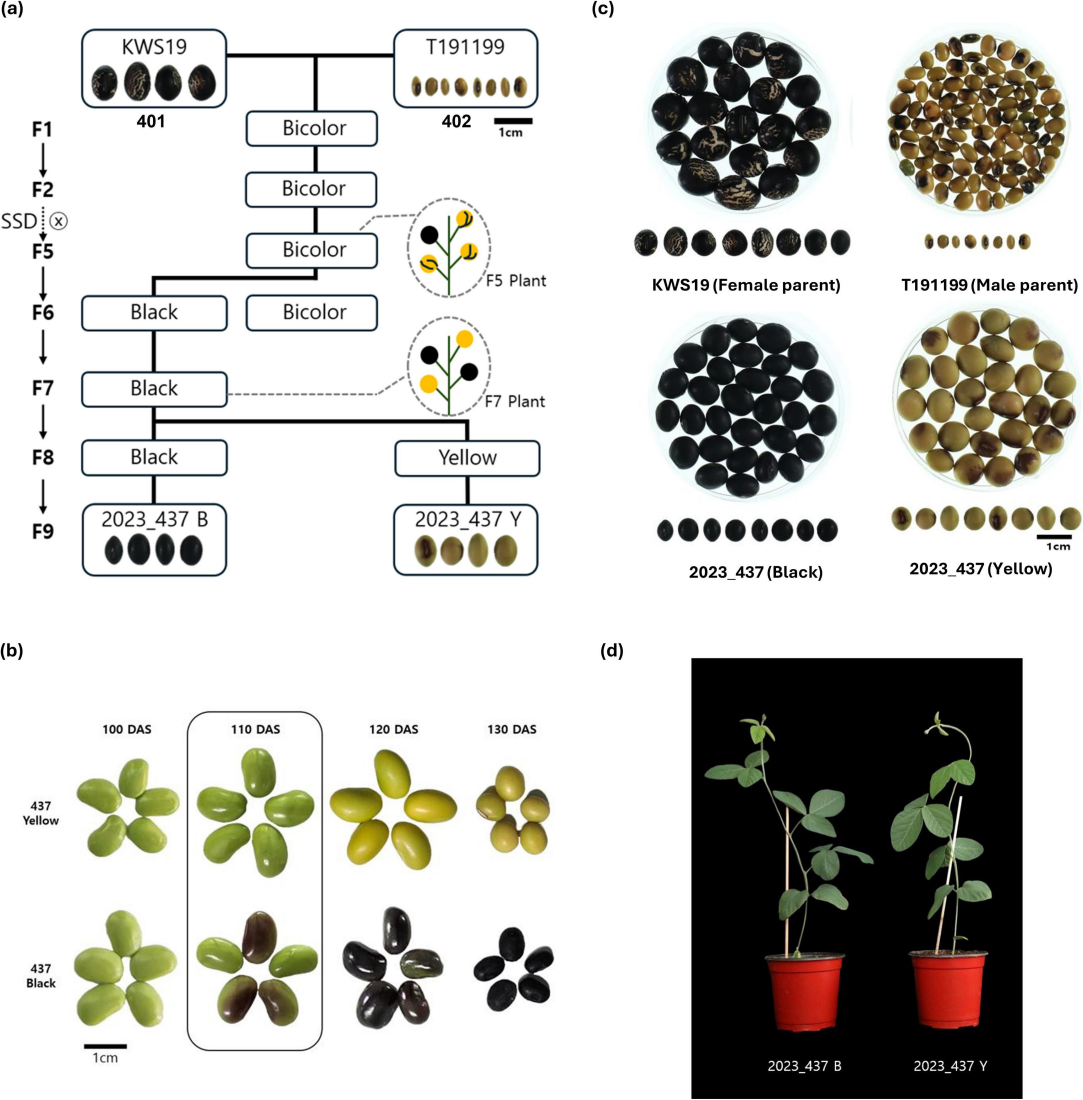
Phenotypic analysis of black and yellow soybean lines. (a) Breeding scheme and recombinant inbred lines (RILs) derived from a cross between a black seed coat Korean landrace (KWS19, female parent) and a yellowish‐brown seed coat semi‐wild type (T191199, male parent). Using single‐seed descent (SSD) from the F1 to F9 generations, resulting in two selected lines exhibiting black (2023_437B) and yellow (2023_437Y) seed coats, respectively. (b) Changes in seed coat color of black (2023_437B) and yellow (2023_437Y) soybean lines during seed development from 100 to 130 days after sowing (DAS). The black soybean line exhibited distinct pigment accumulation starting at 110 DAS, whereas the yellow line maintained a pale‐yellow color throughout maturation. (c) Mature seed phenotypes of the two parental lines (KWS19 and T191199) and the selected F9 progeny (2023_437B and 2023_437Y), illustrating seed coat color variation. (d) Early vegetative stage phenotypes of black (2023_437B) and yellow (2023_437Y) soybean lines, showing similar growth characteristics aside from seed coat color.

To investigate phenotypic and metabolic traits associated with seed coat color, an RIL population was developed from a cross between the Korean black‐seeded landrace KWS19 (female parent) and the tawny, yellowish‐brown semi‐wild accession T191199 (male parent) (Figure 1a). The F1 plants exhibited a bicolored seed coat, indicating segregation of pigmentation alleles from both parents. Successive generations were grown to F5 by single‐seed descent (SSD), after which the F5 plant was selected. Selfing this plant to the F6 generation segregated progeny into bicolor and uniformly black seed coats and continued SSD of black‐seeded plants led to further segregation into black and yellow seed coats at F8. Final selection of uniform plants in F9 yielded two genetically fixed lines with contrasting testa pigmentation: a black‐seeded line (2023_437B) and a yellow‐seeded line (2023_437Y) (Figure 1a).

The two parental cultivars and the F9 lines were grown at the Kangwon National University experimental field (37.9534° N, 127.7459° E) under identical agronomic management for a growth period of approximately 140 days. Seed coat color development was monitored at 10‐day intervals from 100 to 130 days after sowing (DAS). Both lines produced green immature seeds at 100 DAS, but by 110 DAS, the black line 2023_437B showed pronounced onset of dark pigment deposition in the seed coat, whereas 2023_437Y remained uniformly light green to pale yellow (Figure 1b). Between 120 and 130 DAS, seeds of 2023_437B progressively darkened to a fully black testa, whereas seeds of 2023_437Y matured to a stable yellow coat without visible dark mottling (Figure 1b,c). Seeds at 110 DAS, when the divergence in pigmentation first became clearly evident, were harvested for RNA‐seq analysis, and fully mature seeds at physiological maturity were used for detailed metabolite profiling. Aside from testa color, the two F9 lines displayed highly similar early vegetative growth and plant architecture, indicating that they constitute a near‐isogenic pair suitable for dissecting the molecular and metabolic basis of seed coat color differences (Figure 1d).

### 3.2 Metabolite Profiles and Antioxidant Activities in Parental and F9 Black and Yellow Soybean Seeds

**FIGURE 2:**
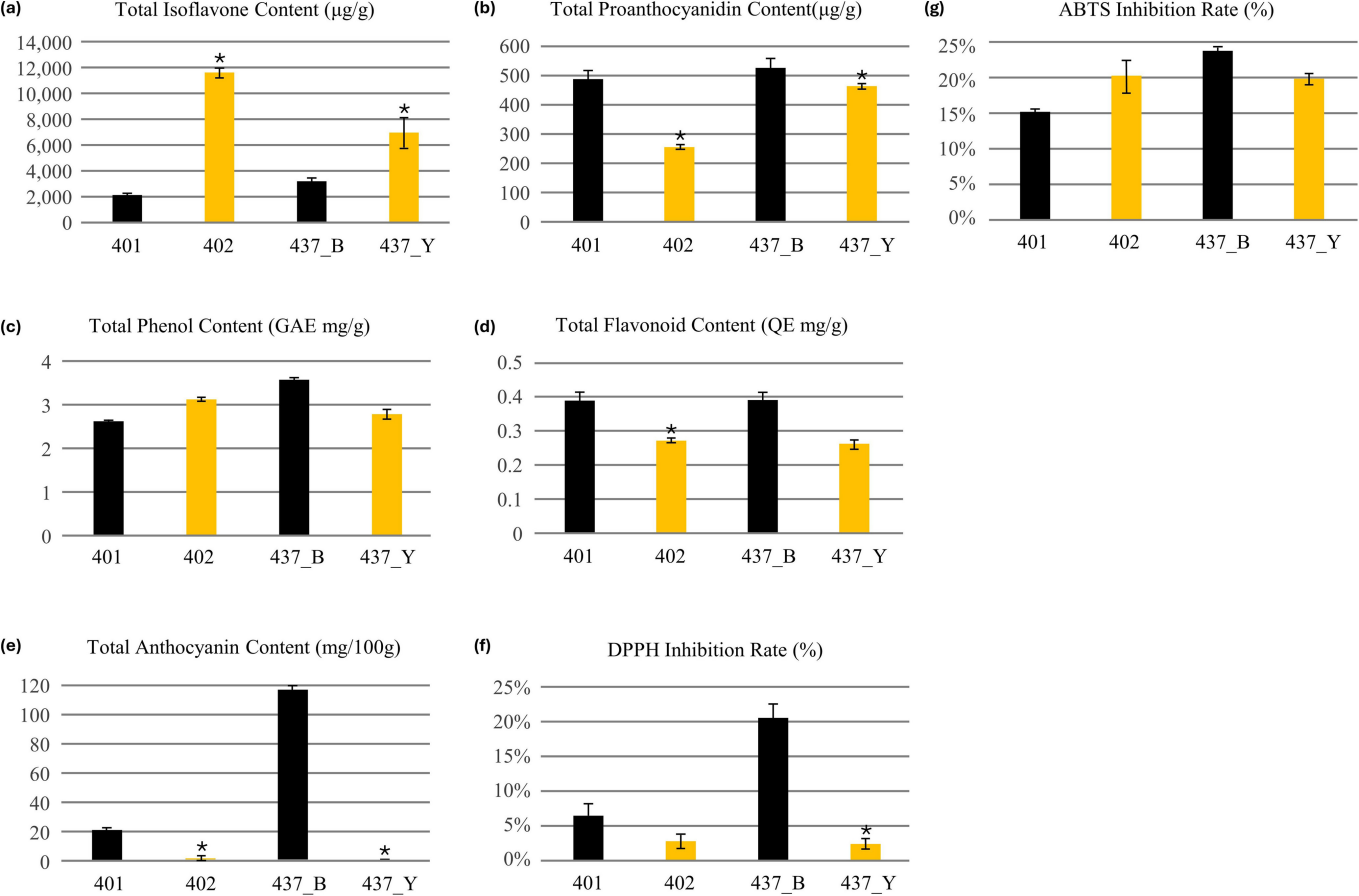
Major metabolite contents and antioxidant activities of seeds from parental and F9 black and yellow soybean lines. Total isoflavone content (TIC) (a), total proanthocyanidin content (TPAC) (b), total phenol content (TPC) (c), total flavonoid content (TFC) (d), total anthocyanin content (TAC) (e), and antioxidant activities—DPPH (f) and ABTS radical scavenging (g) were measured in parental lines (401: KWS19, 402: T191199) as well as in F9 progeny black (437_B) and yellow (437_Y) soybean seeds. Data represent mean ± standard deviation of three biological replicates. Statistical significance between black and yellow lines was assessed by Student's *t*‐test (*p* < 0.05). The F9 black line (437_B) showed high anthocyanin and proanthocyanidin content, as well as antioxidant activity. In contrast, the F9 yellow line (437_Y) exhibited relatively high isoflavone content, reflecting reduced anthocyanin synthesis. Antioxidant activity was strongly correlated with phenolic and flavonoid contents.

Total isoflavone content (TIC) was highest in the yellow parental line 402, followed by the yellow F9 line 437_Y, whereas the black parental line 401 and black F9 line 437_B contained substantially lower levels. This indicates that the yellow‐seeded backgrounds accumulate more isoflavones than the black‐seeded counterparts (Figure 2a). TPAC showed the opposite trend; both black seeds (401 and 437_B) displayed higher TPAC than their yellow counterparts, with 437_B exhibiting the greatest accumulation, consistent with enhanced PA biosynthesis in black testa (Figure 2b). TPC varied within a narrower range but tended to be slightly higher in the F9 black line 437_B than in the parents, whereas 437_Y showed intermediate values (Figure 2c). TFC followed a similar pattern, with higher QE‐based values in 401 and 437_B than in 402 and 437_Y, supporting enrichment of flavonoid constituents in black seeds (Figure 2d).

TAC was strongly genotype dependent. The F9 black line 437_B showed a dramatic increase compared with all other lines, whereas 401 contained only modest levels and both yellow lines (402, 437_Y) had almost negligible anthocyanins. This sharp contrast in TAC parallels the visual pigmentation differences (Figure 2e). Antioxidant activity assays were consistent with these metabolite trends. In the DPPH radical scavenging assay, 437_B showed the highest inhibition rate, followed by 401, whereas both yellow lines exhibited very low activity (Figure 2e). In the ABTS assay, inhibition was also elevated in 437_B and, to a lesser extent, in 402 and 437_Y relative to 401, suggesting contributions from both anthocyanins/PA and isoflavones (Figure 2f). Collectively, these data indicate that the F9 black line combines high PA and anthocyanin levels with superior antioxidant capacity, whereas the yellow lines are characterized by higher isoflavone but lower flavonoid‐derived pigments. The significance of correlation coefficients was evaluated using a two‐tailed Student's *t*‐test (*p* < 0.05).

Correlation analysis among the seven metabolites and antioxidant indicators revealed distinct association patterns. TIC showed strong negative correlations with both TFC and TPAC, indicating a trade‐off between isoflavone accumulation and other flavonoid‐derived compounds. In contrast, TPC was strongly and positively correlated with DPPH and ABTS scavenging activities and with TAC, linking higher phenolic levels to enhanced antioxidant capacity and pigment accumulation. TFC also correlated positively with DPPH activity, anthocyanins, and PAs, while DPPH activity exhibited particularly strong associations with anthocyanins and, to a lesser extent, PA, supporting their key contribution to overall antioxidant activity (Table S1).

### 3.3 DEG Analysis

To uncover the genetic basis of anthocyanin accumulation associated with seed coat pigmentation, RNA‐seq was conducted on seeds harvested at 110 DAS, when clear color divergence between black and yellow lines was visible. A total of ~146.11 million raw reads were generated from 86,256 annotated genes in the soybean reference genome (cv. Williams 82). After quality filtering, ~139.16 million clean reads (94.85%) were retained for analysis. Mapping rates were 90.5% and 92.9% for black and yellow samples, respectively (Table 1). Genes with at least one mapped read were considered expressed, resulting in 28,585 and 29,899 genes identified in black and yellow lines, respectively. To identify DEGs, gene expression was normalized using fragments per kilobase of transcript per Million mapped reads (FPKM), thresholds of |log_2_FoldChange| ≥ 2, *p* ≤ 0.05, and *q* ≤ 0.05. In total, 227 DEGs were identified, with 110 genes upregulated in black soybeans and 117 downregulated in yellow soybeans.

**TABLE 1:** Summary of RNA‐seq analysis and differentially expressed genes (DEGs) between black and yellow soybeans.

Total raw reads	Yellow	Black
	76,006,564	70,107,834
Clean reads	72,394,678	66,769,282
Mapping rate (%)	92.9%	90.5%
Expressed genes (FPKM > 0)	29,899	28,585
DEGs	—	227
Upregulated genes	117	110

### 3.4 GO and Functional Enrichment Analysis

**FIGURE 3:**
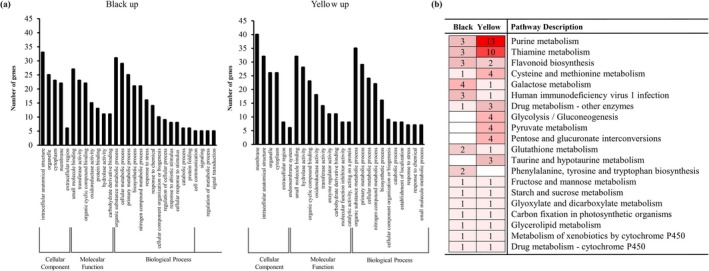
Functional enrichment analysis of differentially expressed genes (DEGs) between black and yellow soybean seed coats. (a) Gene Ontology (GO) enrichment analysis of DEGs upregulated in black and yellow soybean seed coats. Shown are functional annotations categorized by cellular component, molecular function, and biological process. (b) KEGG pathway enrichment analysis comparing DEGs significantly enriched in black and yellow soybean seed coats. Numbers indicate the DEGs involved in each pathway, and darker red shades represent a higher number of enriched genes.

The GO enrichment analysis of DEGs revealed distinct functional trends between black and yellow seed coats (Figure 3). DEGs were predominantly assigned to cellular component categories such as membrane, intracellular anatomical structure, and organelle, indicating that pigmentation differences are associated with changes in membrane and organelle localized processes. Within the molecular function category, terms including oxidoreductase activity, transmembrane transporter activity, and cation binding were strongly represented in both groups, reflecting the importance of redox reactions and transport processes in pigment biosynthesis and accumulation. In the biological process category, DEGs from black seed coats were particularly enriched in carbohydrate metabolic process, response to stimulus, and secondary metabolic process, whereas yellow seed coats showed higher counts in single‐organism metabolic process and cellular metabolic process, suggesting genotype‐specific modulation of primary and secondary metabolism linked to color formation (Figure 3a).

KEGG pathway enrichment further highlighted metabolic pathways that differ between black and yellow seed coats (Figure 3b). Several pathways, including purine metabolism, thiamine metabolism, flavonoid biosynthesis, cysteine and methionine metabolism, glycolysis/gluconeogenesis, pyruvate metabolism, and pentose and glucuronate interconversions, were more highly represented in yellow upregulated DEGs than in black. This indicates enhanced primary and cofactor metabolism in yellow seeds. The pathway analysis indicated clear pathway‐level divergence between lines, notably in flavonoid biosynthesis, as well as differences in purine and thiamine metabolism (Figure 3b). These metabolite and antioxidant data indicate that black seed coats are characterized by strong accumulation of anthocyanins and PAs, which confer higher radical scavenging activity, whereas yellow seed coats largely lack these pigments.

### 3.5 Functional Categorization of Genes in Flavonoid Biosynthesis and Seed Coat Color

Among the 227 DEGs, several functional categories were represented, including flavonoid biosynthetic enzymes (5 genes), laccase (*LAC*), MYB transcription factors (3 genes), cytochrome P450 enzymes (*CYP450*; 7 genes), UDP‐glycosyltransferases (*UGT*; 2 genes), glutathione *S*‐transferase (*GSTF*), and small heat shock proteins (*sHSPs*; 7 genes) (Table 2). We observed marked differences in the expression of key flavonoid pathway genes. Flavanone 3‐hydroxylase (*F3H*; *Glyma.09G243500*), anthocyanidin synthase (*ANS*; *Glyma.01G214200*, *Glyma.11G027700*), genes encoding enzymes that catalyze late steps in anthocyanin biosynthesis (Kim et al. 2008), were significantly upregulated in black soybeans (log_2_FoldChange: 3.24, 2.33, and 3.92) (Table 2). In contrast, leucoanthocyanidin reductase (*LAR*; *Glyma.10G204800*, *Glyma.20G185700*), encoding the enzyme that diverts flux toward PA formation (Bogs et al. 2005), showed higher expression in yellow seed compared to black seed soybean (log_2_FoldChange: −5.11, −2.75) (Table 2).

**TABLE 2:** RNA‐seq analysis of selected differentially expressed genes (DEGs) between black and yellow soybeans. Positive log_2_Fold‐Change values indicate higher gene expression in black soybean, whereas negative values indicate higher expression in yellow soybean. *Arabidopsis* homologs were identified based on BLAST analysis.

Type	*Glycine max*		*Arabidopsis thaliana*		log_2_Fold‐Change
	Accession no.	BLAST result	Accession no,	BLAST result	
Flavonoid biosynthesis genes	*Glyma.09G243500*	Flavanone 3‐dioxygenase (*F3H*)	*AT3G60290*	N/A	3.92
	*Glyma.01G214200*	Anthocyanidin synthase (*ANS*)	*AT4G22880*	LDOX, TT18, TDS4, ANS	3.24
	*Glyma.11G027700*	Leucoanthocyanidin dioxygenase (*ANS*/*LDOX*)	*AT4G22880*	LDOX, TT18, TDS4, ANS	2.33
	*Glyma.20G185700*	Leucoanthocyanidin reductase (*LAR*)	*AT1G75290*	N/A	−2.75
	*Glyma.10G204800*	Leucoanthocyanidin reductase (*LAR*)	*AT1G75290*	N/A	−5.11
LAC	*Glyma.11G233400*	Laccase‐5 (*LAC5*)	*AT2G40370*	LAC5	4.55
MYB	*Glyma.09G235100*	R2R3 MYB transcription factor	*AT1G66370*	MYB113	4.31
	*Glyma.10G236400*	Transcription factor MYB17	*AT3G61250*	MYB17	3.20
	*Glyma.16G023000*	Transcription factor MYB92	*AT5G49330*	PFG3, MYB111, ATMYB111	1.95
CYP	*Glyma.07G267100*	CYP71D8‐like	*AT3G26300*	CYP71B34	2.62
	*Glyma.03G030800*	CYP83B1	*AT4G31500*	RNT1, RED1, SUR2, ATR4, CYP83B1	2.45
	*Glyma.20G114200*	Cinnamate 4‐hydroxylase (*C4H*)	*AT2G30490*	REF3, CYP73A5, C4H, ATC4H	−3.13
	*Glyma.20G189600*	Geraniol 8‐hydroxylase	*AT2G45550*	CYP76C4	−3.17
	*Glyma.01G179400*	CYP71D8‐like	*AT3G26300*	CYP71B34	−5.85
	*Glyma.13G285300*	CYP82A2	*AT4G31940*	CYP82C4	−5.98
	*Glyma.01G135200*	CYP82A4	*AT4G31940*	CYP82C4	−6.30
UGT	*Glyma.13G289000*	UDP‐glycosyltransferase 79B30‐like	*AT5G54010*	UGT79B6	6.04
	*Glyma.08G066800*	UDP‐glucose/flavonoid 3‐*O*‐glucosyltransferase	*AT5G17050*	UGT78D2	2.37
GSTF	*Glyma.18G043700*	Glutathione *S*‐transferase F11‐like	*AT3G03190*	GSTF11, ATGSTF6, ATGSTF11	6.04
HSP	*Glyma.06G134900*	Small heat shock protein, chloroplastic‐like	*AT4G27670*	HSP21	4.79
	*Glyma.08G068800*	Class I heat shock protein	*AT1G07400*	HSP17.8	3.37
	*Glyma.19G011400*	Low molecular weight heat shock protein Hsp22.3 precursor	*AT4G10250*	HSP22	2.78
	*Glyma.08G068700*	HSP20‐like chaperone protein	*AT1G07400*	HSP17.8	2.59
	*Glyma.16G012000*	17.4 kDa Class III heat shock protein	*AT1G54050*	HSP17.4B	2.29
	*Glyma.08G212000*	26.5 kDa heat shock protein, mitochondrial‐like	*AT1G54050*	HSP26.5	2.22
	*Glyma.13G175700*	17.5 kDa Class I heat shock protein‐like protein	*AT1G07400*	HSP17.8	2.11

R2R3‐MYB transcription factors (*Glyma.09G235100*, *Glyma.10G236400*, *Glyma.16G023000*) known to regulate flavonoid biosynthesis (Zhong et al. 2020) were upregulated in black soybeans (Table 2). Most of the cytochrome P450 encoding genes (*Glyma.20G114200*, *Glyma.20G189600*, *Glyma.01G179400*, *Glyma.13G285300*, and *Glyma.01G135200*) were found to be upregulated in yellow soybean seeds (Table 2). The homologs of these genes are related to the oxidative tailoring of phenylpropanoid‐derived compounds (Werck‐Reichhart 1995). UDP‐glycosyltransferase genes (*Glyma.08G066800*, *UGT79B6*; *Glyma.13G289000*) encoding enzymes that glycosylate and stabilize anthocyanidins (Fukuchi‐Mizutani et al. 2003) were upregulated in black soybeans (log_2_FoldChange: 2.37 and 6.04). Other genes specifically upregulated in black seed coats included *GSTF11* (*Glyma.18G043700*), *LAC5* (*Glyma.11G233400*), and several small heat‐shock protein genes (*Glyma.06G134900*, *Glyma.08G068800*, *Glyma.19G011400*) (Table 2). These genes encode proteins that have been reported to function in anthocyanin transport, PA polymerization, and stress‐responsive regulation, respectively.

### 3.6 Validation of Key Genes by RT‐qPCR

**FIGURE 4:**
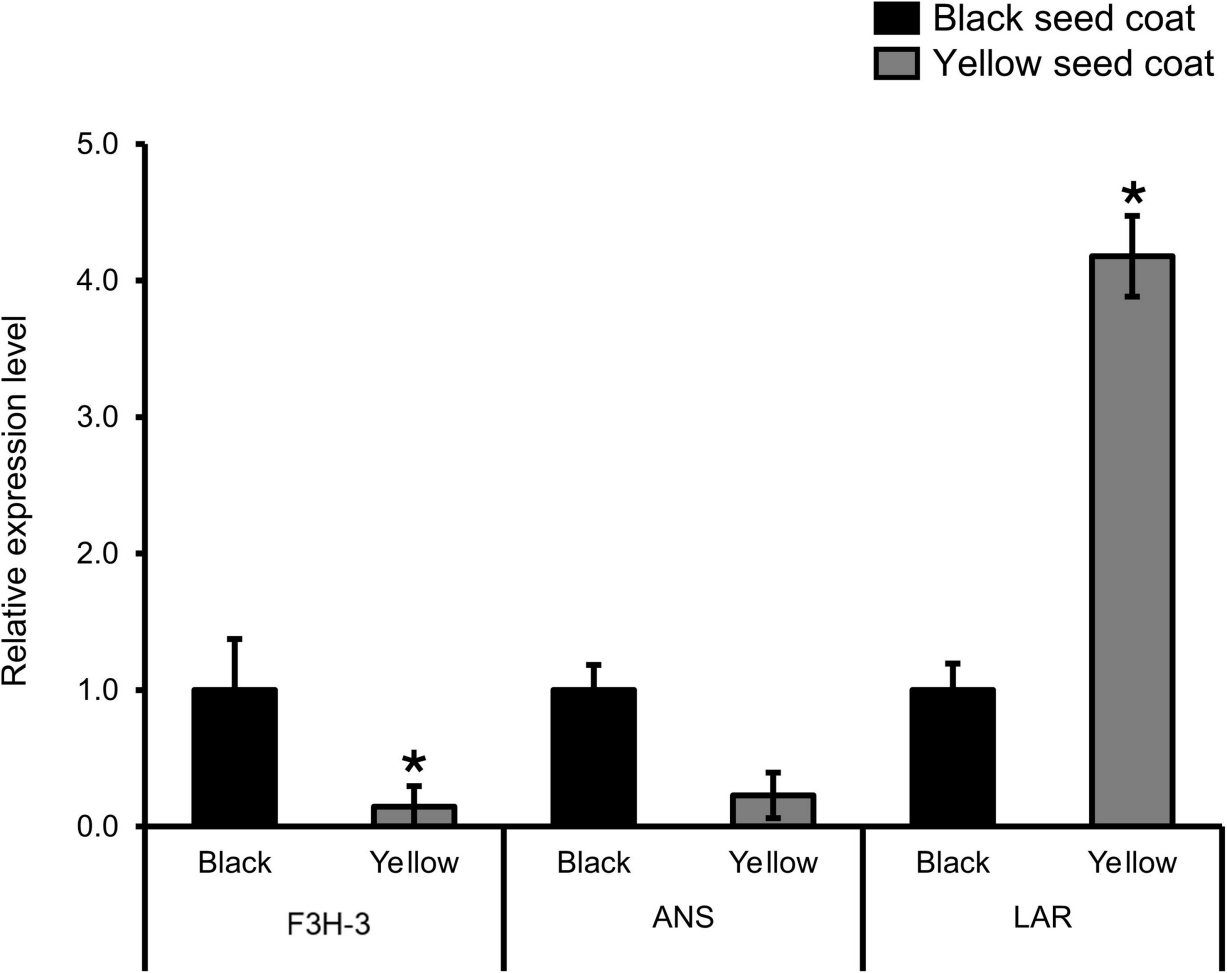
RT‐qPCR analysis of selected genes. The expression patterns of *F3H‐3*, *ANS*, and *LAR* in black and yellow seed coats. The black seeds were used as a control. The data are the mean values of three biological replicates, and the error bars indicate the SDs. Asterisks indicate statistically significant differences between the corresponding samples and their controls (*p* < 0.01, Student's *t*‐test). *Act11* was used as the internal control, and the relative expression levels are shown as fold values.

The expression of three representative flavonoid pathway genes *F3H‐3* (*Glyma.09G243500*), *ANS* (*Glyma.01G214200*), and *LAR* (*Glyma.10G204800*) was quantified in black and yellow seed coats using RT‐qPCR (Figure 4). The expression patterns were consistent with the transcriptome data; *F3H‐3* and *ANS* showed markedly higher transcript levels in black seed coats than in yellow seed coats, supporting their role in promoting anthocyanin biosynthesis in the pigmented testa. In contrast, *LAR* expression was strongly elevated in yellow seed coats compared with black seed coats, in agreement with the predicted shift of metabolic flux toward PA biosynthesis in yellow seeds. Together, these RT‐qPCR results validate the reliability of the RNA‐seq. The differential regulation of these flavonoid biosynthetic genes contributes to the variation in seed coat color between the two soybean genotypes.

## 4 Discussion

This study integrates metabolite measurements with transcriptomics to elucidate molecular processes associated with seed coat color variation between black and yellow soybeans. RNA‐seq analysis was conducted at a single developmental stage (110 DAS), when pigmentation differences were already apparent. Seed coat pigmentation differences primarily reflect contrasting metabolic flux through anthocyanin versus isoflavonoid pathways, as shown by higher TPC, TFC, TAC, and TPAC in black soybeans versus elevated TIC in yellow soybeans. DEG analysis revealed flavonoid biosynthetic genes, glycosyltransferases, transport proteins, polymerization enzymes, R2R3‐MYB transcription factors, and stress‐responsive sHSPs that correlate with anthocyanin and proanthocyanidin accumulation in black seed coats.

### 4.1 Seed Coat–Related Metabolites and Antioxidant Activity

Metabolite profiling revealed distinct biochemical profiles between black and yellow seed coat soybeans. The black‐seeded F9 line (437_B) exhibited significantly higher TPC, TFC, TAC, TPAC, and antioxidant activity (DPPH and ABTS assays) compared to yellow‐seeded lines (437_Y) and the male parent (402) (Figure 2b–g). Conversely, yellow soybeans showed relatively higher TIC (Figure 2a). Previous studies showed phenolic compounds, especially anthocyanins, are essential for seed coat pigmentation, and its absence results in nonpigmented seed coats (Todd and Vodkin 1993), and elevated antioxidant and flavonoids contribute significantly to the prevention and treatment of cardiovascular diseases, cancer, and inflammation (Ganesan and Xu 2017; Wu et al. 2017). These metabolic differences indicate preferential flux toward anthocyanin and PA biosynthesis in black seed coats versus isoflavonoid accumulation in yellow seed coats. Positive correlations between TPC, TFC, TAC, TPAC, and antioxidant activity confirm that anthocyanins and PAs are the primary contributors to the superior radical scavenging capacity of black seed coats, whereas isoflavones make a relatively minor contribution despite their higher abundance in yellow seeds.

### 4.2 Candidate Genes Involved in Seed Coat Pigmentation

#### 4.2.1 Flavanone‐3‐Hydroxylase and Anthocyanidin Synthase Participate in Anthocyanin Biosynthesis

DEG analysis identified candidate genes associated with the flavonoid biosynthesis pathway and seed coat color variation. The flavanone 3‐hydroxylase gene (*F3H*; *Glyma.09G243500*) was significantly upregulated in black soybeans (Figure 4 and Table 2). The elevated *F3H* expression promotes accumulation of anthocyanin pigments in the black seed coat (Cheng et al. 2013). For instance, *F3H* overexpression in tobacco has been found to reduce anthocyanin content (Jiang et al. 2013), whereas white mutants have inactivated *F3H* and show impaired anthocyanin synthesis (Forkmann and Stotz 1984). Similarly, the expression of *ANS* (*Glyma.01G214200*) was markedly upregulated in black seed soybean (Figure 4 and Table 2). A previous study revealed that expression of *ANS* plays an essential role in anthocyanin synthesis in *Arabidopsis* (Pelletier et al. 1997) *ANS* expression correlates with anthocyanin accumulation across species including grape (Gollop et al. 2001), Chinese bayberry (Niu et al. 2010), and strawberry (Almeida et al. 2007), whereas reduced *ANS* expression is associated with anthocyanin deficiency in nonpigmented tissues (Ben‐Simhon et al. 2015). The elevated expression of *F3H* and *ANS* in black soybeans at 110 DAS is therefore consistent with increased anthocyanin biosynthetic flux contributing to seed coat pigmentation observed in this study. However, recent work suggests *ANS* may exhibit context‐dependent regulation in black soybeans (C. Wang et al. 2025), and functional validation will be required to confirm their precise regulatory roles.

#### 4.2.2 LAR and LAC5 Participate in PA Biosynthesis

**FIGURE 5:**
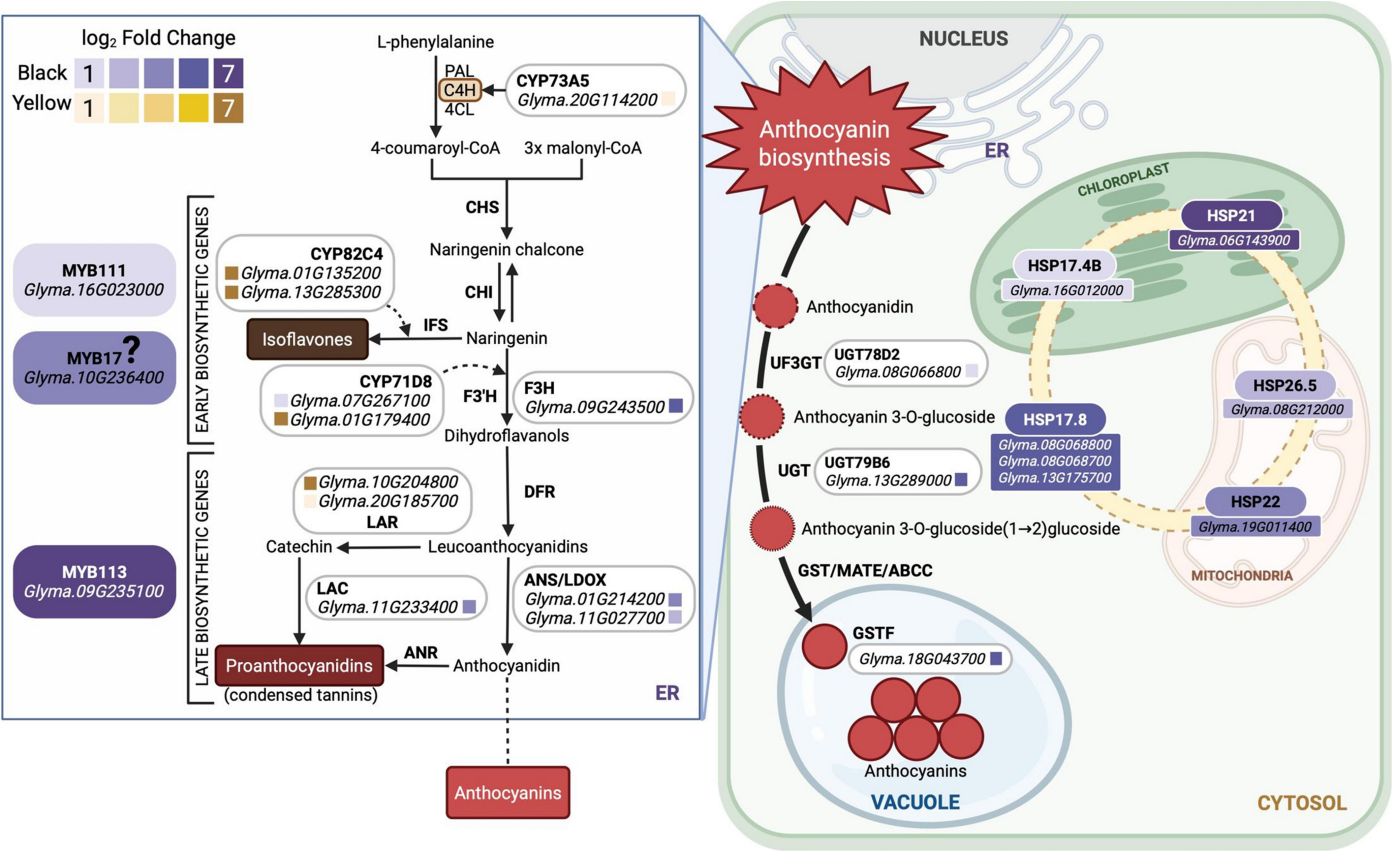
Proposed working model of candidate pathways and genes potentially associated with seed coat coloration based on comparative transcriptomic associations observed at a single developmental stage (110 DAS). This schematic illustrates transcriptomic associations across the phenylpropanoid‐flavonoid pathway from phenylalanine‐derived precursors to anthocyanin and proanthocyanidin (PA) accumulation in vacuoles. Genes upregulated in black seed coats are shown in green and those upregulated in yellow seed coats in brown (color intensity reflects log_2_ fold‐change). Highlighted candidate genes include flavonoid biosynthetic enzymes (*F3H*, *ANS*), glycosyltransferases (*UGT78D2*, *UGT79B6*), transport protein (*GSTF11*), PA polymerization enzyme (*LAC5*; *Glyma.11G233400*), and R2R3‐MYB transcription factors. MYB17 is presented as a candidate regulator (dashed box) based on differential expression, whereas MYB113 represents a well‐characterized anthocyanin activator. Cytochrome P450s (*CYP450*), UDP‐glycosyltransferases (UGTs), and glutathione‐*S*‐transferase (GSTF) are involved in anthocyanin transport and stabilization. *LAC* (*Glyma.11G233400*) showed higher expression in black soybeans, consistent with a possible role in proanthocyanidin polymerization. Small heat shock proteins (sHSPs) are also illustrated, suggesting a potential link between stress responses and pigment accumulation at this developmental stage. Functional studies are required to confirm regulatory roles and temporal dynamics of these candidate genes in seed coat pigmentation.

PAs, or condensed tannins, contribute to seed coat pigmentation and browning (Pourcel et al. 2005). Increased polymerization of PA monomers has been associated with darker tissue coloration and enhanced browning (Hibi and Yanase 2019). Our DEG included *LAR* and *LAC5*, which are linked to PA metabolism in legumes. Expression of *LAR* genes was higher in yellow soybeans, whereas *LAC5* showed elevated transcript levels in black soybeans (Figure 4 and Table 2). Recent research reported that *GmLAR1* influences PA biosynthesis in soybeans, where it competes with the anthocyanin and isoflavone pathways (Chen et al. 2023). However, despite higher *GmLAR1* expression in yellow soybeans in this study, PA content was higher in black soybeans, indicating that *LAR* expression does not directly correlate with PA accumulation. This suggests potential involvement of additional enzymatic processes or complex downstream regulatory mechanisms. In *A. thaliana* , *AtLAC15* promotes PA polymerization during seed coat development, with loss‐of‐function mutants accumulating more soluble PAs and exhibiting delayed browning (Pourcel et al. 2005), whereas *DlLAC5* enhances tissue browning in *Dimocarpus longan* peel through co‐polymerization of PA and lignin monomers (Liu et al. 2024). In this study, *LAC5* exhibited relatively high expression in black seed coats (Table 2), and metabolite profiling indicated higher PA levels in black than in yellow soybeans (Figure 2b). Taken together, these correlative patterns are consistent with a model in which *LAC5* may contribute to PA accumulation and darker seed coat pigmentation in black soybean (Figure 5). However, functional experiments will be required to confirm any direct mechanistic role in PA polymerization or pigment formation.

#### 4.2.3 MYB Transcription Factors Affect Seed Coat Color Determination

R2R3‐MYB transcription factor genes (*Glyma.16G023000*, *Glyma.09G235100*, *Glyma.10G236400*) encoding MYB111, MYB113, and MYB17‐like proteins were significantly upregulated in black soybeans (Table 2). In *Arabidopsis*, MYB113 activates late anthocyanin biosynthetic genes (Stracke et al. 2001), whereas *SmMYB113* in eggplant ( *Solanum melongena* ) to induce anthocyanin biosynthesis (Li et al. 2017). Similarly, soybean R2R3‐MYB transcription factors enhance anthocyanin accumulation (Gillman et al. 2011; Zabala and Vodkin 2014). The elevated expression of *Glyma.09G235100* (encoding MYB113‐like protein) in black soybeans suggests it may contribute to anthocyanin accumulation in the seed coat at 110 DAS. Our findings indicate that MYB111, MYB113, and MYB17, all of which were found to be specifically and highly expressed in black soybeans, are candidate regulators of the flavonoid and anthocyanin biosynthetic pathways in soybean seed coats (Figure 5). These transcriptomic associations provide insights into genetic factors potentially influencing seed coat color and breeding targets for functional soybean cultivars.

#### 4.2.4 CYP450 Genes Determine Seed Coat Color

Cytochrome P450 (CYP450) enzymes play diverse roles in phenylpropanoid and isoflavonoid metabolism (Nelson 2009; Khatri et al. 2022). In this study, expression of *CYP450* genes, *CYP71D8* and *CYP83B1*, was upregulated and *CYP73A5* (encoding C4H), and *geraniol 8‐hydroxylase*, *CYP82A2*, and *CYP82A4* were downregulated in black soybeans and conversely expressed in yellow soybeans (Table 2). The *CYP82A2* and *CYP82A4* genes encode enzymes implicated in isoflavonoid biosynthesis, which is enhanced under stress conditions (Xia et al. 2023). Similarly, CYP82C4 homologs have been associated with flavonoid structural diversification (Khatri et al. 2022). The specific expression of *CYP82C4* in yellow soybeans suggests a potential role in seed coat pigmentation by promoting isoflavonoid synthesis and structural diversification of flavonoids. These results indicate that *CYP450* genes likely contribute to soybean seed coat color via modulation of flavonoid and isoflavonoid pathways (Figure 5). Functionally divergent *CYP450* genes such *as CYP71B34* represent candidate targets for future breeding and molecular studies of pigment regulation.

#### 4.2.5 UGT, GSFT, and sHSPs Affect Seed Coat Color Formation

UDP‐glycosyltransferase genes (*UGT78D2*, *UGT79B6*) encoding 3‐*O*‐glucosyltransferases were significantly upregulated in black seed coat soybeans at 110 DAS (Table 2). These enzymes catalyze the final glycosylation step in anthocyanin biosynthesis, which correlates with pigment accumulation in multiple species (Boss et al. 1996; Cotroneo et al. 2006; Gillman et al. 2011). Previous studies confirm that anthocyanins in black soybeans predominantly exist as 3‐*O*‐glucosides (Choung et al. 2001; Lee et al. 2009). The elevated expression of *UGT78D2* and *UGT79B6* therefore suggests these enzymes may enhance anthocyanin solubility and stability in black seed coats.

The glutathione *S*‐transferase gene (*GSTF11*; *Glyma.18G043700*), homologous to *Arabidopsis* AtGSTF6 and AtGSTF11 (*TT19*), was also upregulated in black seed coats (Table 2). GSTF11 protein facilitates flavonoid vacuolar transport (Harborne and Williams 2000; Grotewold 2006), and suppression of its homolog *MtGSTF7* in *Medicago truncatula* reduces anthocyanin levels (X. Wang et al. 2022). These findings suggest *GSTF11* may contribute to anthocyanin sequestration and proanthocyanidin accumulation in black soybean seeds at this developmental stage (Figure 5). Additionally, *GSTF11* contributes to stress responses, enhancing plant resilience to environmental challenges (Mikhaylova et al. 2021; Musin et al. 2021). RNA‐seq analysis revealed upregulation of several small heat shock protein genes (*sHSPs*) in black seed coats, including *HSP21* (*Glyma.06G134900*) (Table 2). HSP21 has been implicated in chloroplast development, pigment biosynthesis, and plastid‐encoded RNA polymerase function (Zhong et al. 2013), with its expression associated with anthocyanin accumulation in other species (Neta‐Sharir et al. 2005; L. Zhang, Jia, et al. 2023; F. J. Zhang et al. 2024). These correlative expression patterns suggest that sHSPs may link stress responses to pigment accumulation at 110 DAS (Figure 5), although functional validation is required to establish causal roles.

## 5 Conclusions

This study provides transcriptomic and metabolomic insights into seed coat color variation between black and yellow soybeans at 110 DAS. Comparative RNA‐seq analysis identified differential expression of flavonoid biosynthetic genes (*F3H*, *ANS*), glycosyltransferases (*UGT78D2*, *UGT79B6*), transport proteins (*GSTF11*), polymerization enzymes (*LAC5*), R2R3‐MYB transcription factors (*Glyma.09G235100*, *Glyma.16G023000*, *Glyma.10G236400*), and stress‐responsive *sHSPs* specifically associated with black seed coat pigmentation. These correlative expression patterns suggest candidate genetic factors potentially contributing to anthocyanin accumulation and pigmentation differences, although functional validation is required to establish causal regulatory roles. The identified DEGs represent promising markers for soybean breeding programs targeting functional seed coat traits and nutritional enhancement. Future studies employing time‐course transcriptomics, reverse genetics, and biochemical assays will be essential to elucidate the temporal regulatory hierarchies and precise physiological functions of these candidate genes in seed coat color determination and stress‐associated pigmentation.

## Author Contributions

I.Y.C. supervised and designed the research project. K.M. and P.B. performed the research, analyzed data, and wrote the manuscript draft. S.Y.C. performed RNA‐seq analysis. B.‐S.C. conducted bioinformatics analyses. N.‐I.P. and G.L. conducted metabolite analyses. K.‐C.P. and T.Y.U. performed functional analysis. All the authors have read and agreed to the published version of the manuscript.

## Funding

This work was supported by the National Research Foundation of Korea (NRF) grant fund (NRF‐2020R1I1A3052662) and by the Regional Innovation System and Education (RISE) program through the Gangwon RISE Center, funded by the Ministry of Education (MOE) and the Gangwon State (G.S.), Republic of Korea (2025‐RISE‐10‐002).

## Ethics Statement

The authors have nothing to report.

## Consent

The authors have nothing to report.

## Conflicts of Interest

The authors declare no conflicts of interest.

## Peer Review

The peer review history for this article is available in the Supporting Information for this article.

## Supporting information


**Data S1:** Peer review.

**Table S1:** Correlational analysis of metabolite content and antioxidant activity among four soybean samples.
**Table S2:** Primer sequences and Tm values for RT‐qPCR analysis.

## Data Availability

The authors have nothing to report.

## References

[pld370153-bib-0001] Almeida, J. R. , E. D'Amico , A. Preuss , et al. 2007. “Characterization of Major Enzymes and Genes Involved in Flavonoid and Proanthocyanidin Biosynthesis During Fruit Development in Strawberry (*Fragaria × ananassa*).” Archives of Biochemistry and Biophysics 465: 61–71. 17573033 10.1016/j.abb.2007.04.040

[pld370153-bib-0002] Ben‐Simhon, Z. , S. Judeinstein , T. Trainin , et al. 2015. “A White Anthocyanin‐Less Pomegranate (*Punica granatum* L.) Caused by an Insertion in the Coding Region of the Leucoanthocyanidin Dioxygenase (LDOX; ANS) Gene.” PLoS ONE 10: e0142777. 10.1371/journal.pone.0142777. 26581077 PMC4651307

[pld370153-bib-0003] Blois, M. S. 1958. “Antioxidant Determination by the Use of a Stable Free Radical.” Nature 181: 1199–1200.

[pld370153-bib-0004] Bogs, J. , M. O. Downey , J. S. Harvey , A. R. Ashton , G. J. Tanner , and S. P. Robinson . 2005. “Proanthocyanidin Synthesis and Expression of Genes Encoding Leucoanthocyanidin Reductase and Anthocyanidin Reductase in Developing Grape Berries and Grapevine Leaves.” Plant Physiology 139: 652–663. 10.1104/pp.105.064238. 16169968 PMC1255985

[pld370153-bib-0005] Boss, P. K. , C. Davies , and S. P. Robinson . 1996. “Expression of Anthocyanin Biosynthesis Pathway Genes in Red and White Grapes.” Plant Molecular Biology 32: 565–569. 10.1007/BF00019111. 8980508

[pld370153-bib-0006] Campa, A. , E. Perez‐Vega , A. Pascual , and J. J. Ferreira . 2010. “Genetic Analysis and Molecular Mapping of a Quantitative Trait Loci in Common Bean Against *Pythium ultimum* .” Phytopathology 100: 1315–1320. 21062171 10.1094/PHYTO-06-10-0161

[pld370153-bib-0007] Chaturvedi, S. , R. Hemamalini , and S. K. Khare . 2012. “Effect of Processing Conditions on Saponin Content and Antioxidant Activity of Indian Varieties of Soybean (*Glycine max* Linn.).” Annals of Phytomedicine 1: 62–68.

[pld370153-bib-0008] Chen, H. , C. Liu , Y. Li , et al. 2023. “Developmental Dynamic Transcriptome and Systematic Analysis Reveal the Major Genes Underlying Isoflavone Accumulation in Soybean.” Frontiers in Plant Science 14: 1014349. 10.3389/fpls.2023.1014349. 36959940 PMC10027745

[pld370153-bib-0009] Cheng, H. , J. Wang , S. Chu , H. L. Yan , and D. Yu . 2013. “Diversifying Selection on Flavanone 3‐Hydroxylase and Isoflavone Synthase Genes in Cultivated Soybean and Its Wild Progenitors.” PLoS ONE 8: e54154. 23342093 10.1371/journal.pone.0054154 PMC3546919

[pld370153-bib-0010] Choi, H. K. , Y. S. Lim , Y. S. Kim , et al. 2008. “Free‐Radical‐Scavenging and Tyrosinase Inhibition Activities of Cheonggukjang Samples Fermented for Various Times.” Food Chemistry 106: 564–568.

[pld370153-bib-0011] Choung, M. , I. Y. Baek , S. T. Kang , et al. 2001. “Isolation and Determination of Anthocyanins in Seed Coats of Black Soybean (*Glycine max* (L.) Merr.).” Journal of Agricultural and Food Chemistry 49: 5848–5851. 10.1021/jf010550w. 11743773

[pld370153-bib-0012] Cotroneo, P. S. , M. P. Russo , M. Ciuni , G. R. Recupero , and A. R. Lo Piero . 2006. “Quantitative Real‐Time Reverse Transcriptase‐PCR Profiling of Anthocyanin Biosynthetic Genes During Orange Fruit Ripening.” Journal of the American Society for Horticultural Science 131: 537–543.

[pld370153-bib-0013] Dey, A. , M. M. Rahman , A. Gupta , N. Yodo , and C. W. Lee . 2023. “Performance Study on 3D‐Printed Bioplastic Pots From Soybean By‐Products.” Sustainability 15: 10535. 10.3390/su151310535.

[pld370153-bib-0014] Dong, Q. , B. Jiang , Y. Zhang , et al. 2020. “Inheritance Analysis and Gene Mapping of Brown Seed Coat in Soybean.” Soybean Science 39: 361–369. 10.11861/j.issn.1000-9841.2020.03.0361.

[pld370153-bib-0015] Food and Agriculture Organization of the United Nations (FAO) . 2025. “FAOSTAT: Crops and Livestock Products.” Accessed April 10, 2025. https://www.fao.org/faostat/en/#data/QC.

[pld370153-bib-0016] Forkmann, G. , and G. Stotz . 1984. “Selection and Characterisation of Flavanone 3‐Hydroxylase Mutants of *Dahlia*, *Streptocarpus*, *Verbena* and *Zinnia* .” Planta 161: 261–265. 24253654 10.1007/BF00982923

[pld370153-bib-0017] Fukuchi‐Mizutani, M. , H. Okuhara , Y. Fukui , et al. 2003. “Biochemical and Molecular Characterization of a Novel UDP‐Glucose:Anthocyanin 3′‐O‐Glucosyltransferase, a Key Enzyme for Blue Anthocyanin Biosynthesis, From Gentian.” Plant Physiology 132: 1652–1663. 12857844 10.1104/pp.102.018242 PMC167102

[pld370153-bib-0018] Ganesan, K. , and B. Xu . 2017. “A Critical Review on Polyphenols and Health Benefits of Black Soybeans.” Nutrients 9: 455. 10.3390/nu9050455. 28471393 PMC5452185

[pld370153-bib-0019] Gillman, J. D. , A. Tetlow , J. D. Lee , J. G. Shannon , and K. Bilyeu . 2011. “Loss‐of‐Function Mutations Affecting a Specific *Glycine max* R2R3 MYB Transcription Factor Result in Brown Hilum and Brown Seed Coats.” BMC Plant Biology 11: 155. 10.1186/1471-2229-11-155. 22070454 PMC3229458

[pld370153-bib-0020] Giusti, M. M. , and R. E. Wrolstad . 2001. “Characterization and Measurement of Anthocyanins by UV‐Visible Spectroscopy.” In Current Protocols in Food Analytical Chemistry, edited by R. E. Wrolstad , F1.2.1–F1.2.13. John Wiley & Sons.

[pld370153-bib-0021] Gollop, R. , S. Farhi , and A. Perl . 2001. “Regulation of the Leucoanthocyanidin Dioxygenase Gene Expression in *Vitis vinifera* .” Plant Science 161: 579–588.

[pld370153-bib-0022] Grotewold, E. 2006. “The Genetics and Biochemistry of Floral Pigments.” Annual Review of Plant Biology 57: 761–780. 10.1146/annurev.arplant.57.032905.105248 16669781

[pld370153-bib-0023] Harborne, J. B. , and C. A. Williams . 2000. “Advances in Flavonoid Research Since 1992.” Phytochemistry 55: 481–504. 11130659 10.1016/s0031-9422(00)00235-1

[pld370153-bib-0024] Hibi, Y. , and E. Yanase . 2019. “Oxidation of Procyanidins With Various Degrees of Condensation: Influence on the Color‐Deepening Phenomenon.” Journal of Agricultural and Food Chemistry 67: 4940–4946. 10.1021/acs.jafc.9b02085. 30994340

[pld370153-bib-0025] Hirai, M. Y. , M. Yano , D. B. Goodenowe , et al. 2004. “Integration of Transcriptomics and Metabolomics for Understanding of Global Responses to Nutritional Stresses in *Arabidopsis thaliana* .” Proceedings of the National Academy of Sciences of the United States of America 101: 10205–10210. 15199185 10.1073/pnas.0403218101 PMC454188

[pld370153-bib-0026] Hosamani, J. , M. Dadlani , I. M. Santha , M. B. A. Kumar , and S. R. Jacob . 2013. “Biochemical Phenotyping of Soybean [*Glycine max* (L.) Merrill] Genotypes to Establish the Role of Lipid Peroxidation and Antioxidant Enzymes in Seed Longevity.” Agricultural Research 2: 119–126.

[pld370153-bib-0027] Jiang, F. , J. Y. Wang , H. F. Jia , W. S. Jia , H. Q. Wang , and M. Xiao . 2013. “RNAi‐Mediated Silencing of the Flavanone 3‐Hydroxylase Gene and Its Effect on Flavonoid Biosynthesis in Strawberry Fruit.” Journal of Plant Growth Regulation 32: 182–190.

[pld370153-bib-0028] Joshi, T. , and D. Xu . 2022. “Metabolomics as a Prospective Tool for Soybean (*Glycine max*) Crop Improvement.” Biomolecules 12, no. 9: 287. 36135199 10.3390/cimb44090287 PMC9497771

[pld370153-bib-0029] Kafer, J. M. , M. D. C. Molinari , F. A. Henning , et al. 2023. “Transcriptional Profile of Soybean Seeds With Contrasting Seed Coat Color.” Plants 12: 1555. 10.3390/plants12071555. 37050181 PMC10097363

[pld370153-bib-0030] Khatri, P. , O. Wally , I. Rajcan , and S. Dhaubhadel . 2022. “Comprehensive Analysis of Cytochrome P450 Monooxygenases Reveals Insight Into Their Role in Partial Resistance Against *Phytophthora sojae* in Soybean.” Frontiers in Plant Science 13: 862314. 10.3389/fpls.2022.862314. 35498648 PMC9048032

[pld370153-bib-0031] Kim, J. H. , Y. J. Lee , B. G. Kim , Y. Lim , and J. H. Ahn . 2008. “Flavanone 3β‐Hydroxylases From Rice: Key Enzymes for Flavonol and Anthocyanin Biosynthesis.” Molecules and Cells 25: 312–316. 18413994

[pld370153-bib-0032] Kyle, M. M. , and M. H. Dickson . 1988. “Linkage of Hypersensitivity to Five Viruses With the Locus in *Phaseolus vulgaris* L.” Journal of Heredity 79: 308–311.

[pld370153-bib-0033] Lee, C. , M.‐S. Choi , H.‐T. Kim , et al. 2015. “Soybean [*Glycine max* (L.) Merrill]: Importance as a Crop and Pedigree Reconstruction of Korean Varieties.” Plant Breeding and Biotechnology 3: 179–196.

[pld370153-bib-0034] Lee, J. H. , N. S. Kang , S. O. Shin , et al. 2009. “Characterisation of Anthocyanins in the Black Soybean (*Glycine max* L.) by HPLC‐DAD‐ESI/MS Analysis.” Food Chemistry 112: 226–231. 10.1016/j.foodchem.2008.05.056.

[pld370153-bib-0035] Lee, J. H. , E. Y. Seo , and Y. M. Lee . 2023. “Comparative Investigation on Variations of Nutritional Components in Whole Seeds and Seed Coats of Korean Black Soybeans for Different Crop Years and Screening of Their Antioxidant and Anti‐Aging Properties.” Food Chemistry: X 17: 100572. 10.1016/j.fochx.2023.100572. 36845484 PMC9944501

[pld370153-bib-0036] Li, J. , L. Ren , Z. Gao , et al. 2017. “Combined Transcriptomic and Proteomic Analysis Constructs a New Model for Light‐Induced Anthocyanin Biosynthesis in Eggplant (*Solanum melongena* L.).” Plant, Cell & Environment 40: 3069–3087. 10.1111/pce.13074 28940206

[pld370153-bib-0037] Liu, B. , F. Fang , H. Guan , et al. 2024. “Integrated Function of Proanthocyanidin and Lignin Polymerization Mediated by LAC/PRXs in Pericarp Browning of Longan Fruit.” Postharvest Biology and Technology 207: 112618. 10.1016/j.postharvbio.2023.112618.

[pld370153-bib-0038] Liu, C. , X. Chen , W. Wang , et al. 2021. “Identifying Wild Versus Cultivated Gene‐Alleles Conferring Seed Coat Color and Days to Flowering in Soybean.” International Journal of Molecular Sciences 22: 1559. 10.3390/ijms22041559. 33557103 PMC7913812

[pld370153-bib-0039] Liu, J. , W. Qin , H. Wu , et al. 2017. “Metabolism Variation and Better Storability of Dark‐ Versus Light‐Coloured Soybean (*Glycine max* L. Merr.) Seeds.” Food Chemistry 223: 104–113. 28069115 10.1016/j.foodchem.2016.12.036

[pld370153-bib-0040] Ma, C. , Y. Feng , S. Zhou , et al. 2023. “Metabolomics and Transcriptomics Provide Insights Into the Molecular Mechanisms of Anthocyanin Accumulation in the Seed Coat of Differently Colored Mung Bean (*Vigna radiata* L.).” Plant Physiology and Biochemistry 200: 107739. 10.1016/j.plaphy.2023.107739. 37196373

[pld370153-bib-0041] Medic, J. , C. Atkinson , and C. R. Hurburgh . 2014. “Current Knowledge in Soybean Composition.” Journal of the American Oil Chemists' Society 91: 363–384.

[pld370153-bib-0042] Mikhaylova, E. , E. Khusnutdinov , M. Y. Shein , V. Y. Alekseev , Y. Nikonorov , and B. Kuluev . 2021. “The Role of the GSTF11 Gene in Resistance to Powdery Mildew Infection and Cold Stress.” Plants 10: 2729. 34961200 10.3390/plants10122729 PMC8704923

[pld370153-bib-0043] Moïse, J. A. , S. Han , L. Gudynaitė‐Savitch , D. A. Johnson , and B. L. A. Miki . 2005. “Seed Coats: Structure, Development, Composition, and Biotechnology.” In Vitro Cellular & Developmental Biology: Plant 41: 620–644.

[pld370153-bib-0044] Musin, K. G. , V. V. Fedyaev , and B. R. Kuluev . 2021. “Antioxidant System and Long‐Term Storage of Hairy Roots of Tobacco With Constitutive Expression of Glutathione‐S‐Transferase Gene AtGSTF11.” Russian Journal of Plant Physiology 68: 380–391.

[pld370153-bib-0045] National Food Research Institute (NFRI) . 1990. “Manuals of Quality Characteristic Analysis for Food Quality Evaluation (2).” National Food Research Institute, (in Japanese).

[pld370153-bib-0046] Ncube, E. , K. Mohale , and N. Nogemane . 2022. “Metabolomics as a Prospective Tool for Soybean (*Glycine max*) Crop Improvement.” Current Issues in Molecular Biology 44, no. 9: 4181–4196. 36135199 10.3390/cimb44090287 PMC9497771

[pld370153-bib-0047] Nelson, D. R. 2009. “The Cytochrome P450 Homepage.” Human Genomics 4: 59–65. 19951895 10.1186/1479-7364-4-1-59 PMC3500189

[pld370153-bib-0048] Neta‐Sharir, I. , T. Isaacson , S. Lurie , and D. Weiss . 2005. “Dual Role for Tomato Heat Shock Protein 21: Protecting Photosystem II From Oxidative Stress and Promoting Color Changes During Fruit Maturation.” Plant Cell 17: 1829–1838. 10.1105/tpc.105.031914. 15879560 PMC1143080

[pld370153-bib-0049] Niu, S. S. , C. J. Xu , W. S. Zhang , et al. 2010. “Coordinated Regulation of Anthocyanin Biosynthesis in Chinese Bayberry (*Myrica rubra*) Fruit by a R2R3 MYB Transcription Factor.” Planta 231: 887–899. 20183921 10.1007/s00425-009-1095-z

[pld370153-bib-0050] Pelletier, M. K. , J. R. Murrell , and B. W. Shirley . 1997. “Characterization of Flavonol Synthase and Leucoanthocyanidin Dioxygenase Genes in *Arabidopsis* .” Plant Physiology 113: 1437–1445. 9112784 10.1104/pp.113.4.1437 PMC158268

[pld370153-bib-0051] Pourcel, L. , J. M. Routaboul , L. Kerhoas , M. Caboche , L. Lepiniec , and I. Debeaujon . 2005. “TRANSPARENT TESTA10 Encodes a Laccase‐Like Enzyme Involved in Oxidative Polymerization of Flavonoids in *Arabidopsis* Seed Coat.” Plant Cell 17: 2966–2980. 16243908 10.1105/tpc.105.035154 PMC1276023

[pld370153-bib-0052] Qiu, H. M. , L. Chen , Y. L. Hou , et al. 2021. “Research Progress on Genetic Regulatory Mechanism of Seed Color in Soybean (*Glycine max*).” Acta Agronomica Sinica 47: 2299–2313.

[pld370153-bib-0053] Re, R. , N. Pellegrini , A. Proteggente , A. Pannala , M. Yang , and C. Rice‐Evans . 1999. “Antioxidant Activity Applying an Improved ABTS Radical Cation Decolorization Assay.” Free Radical Biology and Medicine 26: 1231–1237. 10381194 10.1016/s0891-5849(98)00315-3

[pld370153-bib-0054] Song, X. , J. Diao , J. Ji , et al. 2016. “Molecular Cloning and Identification of a Flavanone 3‐Hydroxylase Gene From *Lycium chinense*, and Its Overexpression Enhances Drought Stress in Tobacco.” Plant Physiology and Biochemistry 98: 89–100. 26650932 10.1016/j.plaphy.2015.11.011

[pld370153-bib-0055] Stracke, R. , M. Werber , and B. Weisshaar . 2001. “The R2R3‐MYB Gene Family in *Arabidopsis thaliana* .” Current Opinion in Plant Biology 4: 447–456. 11597504 10.1016/s1369-5266(00)00199-0

[pld370153-bib-0056] Todd, J. J. , and L. O. Vodkin . 1993. “Pigmented Soybean (*Glycine max*) Seed Coats Accumulate Proanthocyanidins During Development.” Plant Physiology 102: 663–670. 10.1104/pp.102.2.663. 12231856 PMC158826

[pld370153-bib-0057] Tuteja, J. H. , S. J. Clough , W. C. Chan , and L. O. Vodkin . 2004. “Tissue‐Specific Gene Silencing Mediated by a Naturally Occurring Chalcone Synthase Gene Cluster in *Glycine max* .” Plant Cell 16: 819–835. 10.1105/tpc.021352. 15064367 PMC412859

[pld370153-bib-0058] U.S. Department of Agriculture (USDA) . 2024. “Oilseeds: World Markets and Trade. World Production, Markets, and Trade Report.” Accessed July 13, 2024. https://fas.usda.gov/data/oilseeds‐world‐markets‐and‐trade‐07122024.

[pld370153-bib-0059] Voysest, O. 2000. Mejoramiento Genético del Frijol (*P. vulgaris* L.): Legado de Variedades de América Latina 1930–1999 (Publicación CIAT No. 321). Centro Internacional de Agricultura Tropical (CIAT).

[pld370153-bib-0060] Wang, C. , P. Fu , T. Sun , et al. 2025. “Identifying Candidate Genes Related to Soybean (*Glycine max*) Seed Coat Color via RNA‐seq and Coexpression Network Analysis.” Genes 16: 44. 39858589 10.3390/genes16010044 PMC11764550

[pld370153-bib-0062] Wang, X. , Y. Liu , L. Ouyang , et al. 2022. “Metabolomics Combined With Transcriptomics Analyses of Mechanism Regulating Testa Pigmentation in Peanut.” Frontiers in Plant Science 13: 1065049. 10.3389/fpls.2022.1065049. 36589085 PMC9800836

[pld370153-bib-0063] Waqas, M. K. , N. Akhtar , R. Mustafa , M. Jamshaid , H. M. S. Kan , and G. Murtaza . 2015. “Dermatological and Cosmeceutical Benefits of *Glycine max* (Soybean) and Its Active Components.” Acta Poloniae Pharmaceutica. Drug Research 72: 3–11. 25850195

[pld370153-bib-0064] Werck‐Reichhart, D. 1995. “Cytochromes P450 in Phenylpropanoid Metabolism.” Drug Metabolism and Drug Interactions 12: 221–243. 8820854 10.1515/dmdi.1995.12.3-4.221

[pld370153-bib-0065] Wu, I. , and A. G. Paice . 2011. “Black Soybean (*Glycine max* L. Merrill) Seeds' Antioxidant Capacity.” In Nuts and Seeds in Health and Disease Prevention, edited by V. Preedy , R. Watson , and V. Patel , 229–236. Elsevier.

[pld370153-bib-0066] Wu, T. , X. Guo , M. Zhang , L. Yang , R. Liu , and J. Yin . 2017. “Anthocyanins in Black Rice, Soybean, and Purple Corn Increase Fecal Butyric Acid and Prevent Liver Inflammation in High‐Fat Diet‐Induced Obese Mice.” Food & Function 8: 3178–3186. 10.1039/C7FO00449D. 28792056

[pld370153-bib-0067] Xia, Y. , C. He , S. Yan , et al. 2023. “New Dual Functional CYP450 Gene Involves in Isoflavone Biosynthesis in *Glycine max* L.” Synthetic and Systems Biotechnology 8: 157–167. 10.1016/j.synbio.2023.01.002. 36714060 PMC9860299

[pld370153-bib-0068] Yang, K. , N. Jeong , J. K. Moon , et al. 2010. “Genetic Analysis of Genes Controlling Natural Variation of Seed Coat and Flower Colors in Soybean.” Journal of Heredity 101: 757–768. 10.1093/jhered/esq078. 20584753

[pld370153-bib-0069] Yang, Y. , T. Zhao , F. Wang , et al. 2023. “Identification of Candidate Genes for Soybean Seed Coat‐Related Traits Using QTL Mapping and GWAS.” Frontiers in Plant Science 14: 1190503. 10.3389/fpls.2023.1190503. 37384360 PMC10293793

[pld370153-bib-0070] Yi, T. G. , Y. R. Park , I. Y. Choi , and N. I. Park . 2019. “Comparison of Metabolite Levels and Antioxidant Activity Among Pepper Cultivars.” Korean Journal of Breeding Science 51: 326–340. 10.9787/KJBS.2019.51.4.326.

[pld370153-bib-0071] Yu, T. , X. Ma , J. Zhang , et al. 2025. “Progress in Transcriptomics and Metabolomics in Plant Responses to Abiotic Stresses.” Current Issues in Molecular Biology 47: 421. 10.3390/cimb47060421. 40699820 PMC12191765

[pld370153-bib-0072] Zabala, G. , and L. O. Vodkin . 2014. “Methylation Affects Transposition and Splicing of a Large CACTA Transposon From a MYB Transcription Factor Regulating Anthocyanin Synthase Genes in Soybean Seed Coats.” PLoS ONE 9: e111959. 10.1371/journal.pone.0111959. 25369033 PMC4219821

[pld370153-bib-0073] Zhang, C. , Y. Zhang , Z. Su , X. Chen , X. Zhang , and H. Liu . 2023. “Integrated Analysis of HSP20 Genes in the Developing Flesh of Peach: Identification, Expression Profiling, and Subcellular Localization.” BMC Plant Biology 23: 663. 10.1186/s12870-023-04621-0. 38129812 PMC10740231

[pld370153-bib-0074] Zhang, F. J. , Z. Y. Li , D. E. Zhang , et al. 2024. “Identification of Hsp20 Gene Family in *Malus domestica* and Functional Characterization of Hsp20 Class I Gene MdHsp18.2b.” Physiologia Plantarum 176: e14288. 10.1111/ppl.14288. 38644531

[pld370153-bib-0075] Zhang, L. , R. Jia , L. Liu , et al. 2023. “Seed Coat Colour and Structure Are Related to the Seed Dormancy and Overwintering Ability of Crop‐to‐Wild Hybrid Soybean.” AoB Plants 15: plad081. 38090392 10.1093/aobpla/plad081 PMC10712219

[pld370153-bib-0076] Zhong, C. , Y. Tang , B. Pang , et al. 2020. “The R2R3‐MYB Transcription Factor GhMYB1a Regulates Flavonol and Anthocyanin Accumulation in *Gerbera hybrida* .” Horticulture Research 7: 78. 32435501 10.1038/s41438-020-0296-2 PMC7237480

[pld370153-bib-0077] Zhong, L. , W. Zhou , H. Wang , et al. 2013. “Chloroplast Small Heat Shock Protein HSP21 Interacts With Plastid Nucleoid Protein pTAC5 and Is Essential for Chloroplast Development in *Arabidopsis* Under Heat Stress.” Plant Cell 25: 2925–2943. 10.1105/tpc.113.111229. 23922206 PMC3784589

