# Supplementary material for: Transcriptomic and Metabolic Insight Into Flavonoid Biosynthesis Underlying Black and Yellow Seed Coat Color Variation in Soybean ( Glycine max )
Source: Plant Direct. 2026 Feb 20;10(2):e70153. doi: 10.1002/pld3.70153 (PMC12921420; doi:10.1002/pld3.70153)
Supplement: Supplementary file 2 — Table S1: Correlational analysis of metabolite content and antioxidant activity among four soybean samples. Table S2: Primer sequences and Tm values for RT‐qPCR analysis. [file PLD3-10-e70153-s001.docx]

Supplementary Table S1. Correlational analysis of metabolite content and antioxidant activity among four soybean samples

|  | TIC | TPC | TFC | DPPH | ABTS | TAC |
| --- | --- | --- | --- | --- | --- | --- |
| TIC |  |  |  |  |  |  |
| TPC | 0.059 |  |  |  |  |  |
| TFC | −0.813^**^ | 0.260 |  |  |  |  |
| DPPH rate | −0.560 | 0.771^**^ | 0.749^**^ |  |  |  |
| ABTS rate | 0.200 | 0.852^**^ | −0.041 | 0.568 |  |  |
| TAC | −0.547 | 0.793^**^ | 0.708^**^ | 0.988^**^ | 0.611^*^ |  |
| TPAC | −0.894^**^ | 0.033 | 0.649^*^ | 0.593^*^ | 0.039 | 0.570 |

Pearson correlation coefficients were calculated for: total isoflavone content (TIC), total phenolic content (TPC), total flavonoid content (TFC), two measurements of antioxidant activity (i.e., DPPH, ABTS), total anthocyanin content (TAC), and total proanthocyanidin content (TPAC) as measured in parental (401, 402) and F9 lines (437_B, 437_Y). Statistical significance was indicated as **p* < 0.05 and ***p* < 0.01. Negative correlations indicate that higher isoflavone content is associated with lower flavonoid and proanthocyanidin content, whereas positive correlations suggest that increased phenolic, flavonoid, and anthocyanin content are associated with higher antioxidant activity.

Supplementary Table S2. Primer sequences and Tm values for RT-qPCR analysis

| Gene ID | Forward | Reverse | Gene Symbol |
| --- | --- | --- | --- |
| *Glyma.01G214200* | AACCCGCTGATTATATTGAGGTC | CTTCCTTCTCCAACCTCCGTC | *ANS* |
| *Glyma.09G243500* | TGTCGGTGATATGATCCAGGTTT | TCTTCCAAGGGCTTCACATCA | *F3H3* |
| *Glyma.10G204800* | TCATGGCAATGTCAAAGCTTACTT | GTCCTGGCATCATCAACAACC | *LAR* |
| *Glyma.02G091900* | ATTTTGACTGAGCGTGGTTATTCCTT | GAGCTGGTCCTGGCTGTCTCC | *Act11* |
