# Supplementary material for: Transcriptomic and Metabolic Insight Into Flavonoid Biosynthesis Underlying Black and Yellow Seed Coat Color Variation in Soybean ( Glycine max )
Source: Plant Direct. 2026 Feb 20;10(2):e70153. doi: 10.1002/pld3.70153 (PMC12921420; doi:10.1002/pld3.70153)
Supplement: Supplementary file 1 — Data S1: Peer review. [file PLD3-10-e70153-s002.pdf]

Transcriptomic and Metabolic Insight into Flavonoid Biosynthesis Underlying Black and Yellow Seed Coat Color Variation in Soybean (Glycine max)

Ik-Young Choi, Kahee MOON , Dr. Prakash Basnet , Seung Young Choi , Dr. Beom-Soon Choi , Nam-il Park , Kyong-Cheul Park , Grace Lachica , Taeyoung Um

Decision Letter Round 1:

November 26, 2025

Prof. Ik-Young Choi

Kangwon National University

Chuncheon

Korea (South), Republic of

RE: Transcriptomic and Metabolic Insight into Flavonoid Biosynthesis Underlying Seed Coat Color Variation in Soybean (Glycine max)

Dear Dr. Choi:

Thank you for submitting to Plant Direct. All required reviews have been returned and we have now finished our evaluation of your manuscript. In light of the reviewers' and editor's comments, further revisions are needed before the paper can be accepted for publication in Plant Direct.

Please view the editors' and reviewers' comments below and use their suggestions as a guide while you work on your revision.

When uploading the revised version of this article, please be sure to include the following:

-A word document that contains your response to the reviewers. You should respond to each reviewer comment and note the changes made to the manuscript. If you do not agree with a reviewer's comment and choose not to make a suggested revision, please explain why. Please try to provide as complete an answer as possible to each reviewer's criticisms in the "response to reviewers" section.

-A tracked changes document

- A clean version of the latest version of the manuscript

To upload your revision, please click the link below.

https://plantdirect.msubmit.net/cgi-bin/main.plex?el=A3Lr7pj6A5nPI3I7A9ftd7uvTLdjPSG4Sifp6rTgFIQZ

In order to provide as timely a service as possible, we ask that your revision is resubmitted within three months after receipt of this request. If an extension is needed, please send a request, along with a brief explanation, to the editorial office at plantdirect@wiley.com .

Thank you very much for giving us an opportunity to review your work. I look forward to receiving the next version.

Sincerely,

Gustavo MacIntosh

--- Reviewer comments:

Reviewer #1:

All comments mentioned in attachment file

Reviewer #2:

Review Comments to the Authors (with line numbers)

Thank you for submitting this manuscript. The topic is relevant and the dataset has potential value; however, several essential issues regarding methodological transparency, statistical reporting, and clarity of interpretation need to be addressed to ensure reproducibility and alignment with Plant Direct's sound-science criteria. Below are detailed comments.

Major Comments

1. Biological replicate number (n) missing for metabolite assays and RT-qPCR assays

Location:

- RESULTS, L111-117

- Figure 2 legend

- Figure 4 legend

- MATERIALS AND METHODS, Metabolite Analysis & RT-qPCR sections

Figure 2 reports mean {plus minus} SD values, but the number of biological replicates (n) is not stated in the figure, its legend, or the MATERIALS AND METHODS.

Without n, the robustness and reproducibility of the metabolite data cannot be evaluated.

Please specify the biological replicate number for each measurement, and consider providing replicate-level data in supplementary materials.

In addition to Fig. 2, Fig. 4 also lacks information on the number of biological replicates (n).

Even though error bars are shown (standard error), the absence of n makes it impossible to assess the robustness of the RT-qPCR validation.

Please specify the number of biological replicates used for each qPCR assay.

2. Sample extraction and preparation methods insufficiently described

Location: Metabolite Analysis section, L493-498

Although several published protocols are cited, the manuscript does not describe:

- seed grinding method,
- extraction solvent(s), ratio, or volume,
- extraction temperature, time, or equipment,
- centrifugation/filtration steps.

These steps are essential for reproducibility, even when established methods are cited.

Please include a concise but complete description of extraction procedures.

3. Analytical conditions for HPLC, spectrophotometry, and radical scavenging assays are insufficiently described

Location: Metabolite Analysis section, L493-498

The manuscript does not provide the essential analytical conditions required for reproducing any of the metabolite assays. Missing information includes:

- Instrument information (manufacturer/model of HPLC system, spectrophotometer/microplate reader, etc.)
- HPLC conditions (column type/dimensions, mobile phase, gradient, flow rate, wavelength, temperature)
- Spectrophotometric assay conditions (wavelengths, incubation times, standard curves, calculation methods)
- DPPH/ABTS assay conditions (reagent concentrations, reaction times, wavelengths, % inhibition formula)

Because these details are absent, the assays cannot be reproduced.

Please provide the minimum essential analytical conditions to ensure reproducibility.

4. Statistical claims ("significantly higher", "strongly correlated") are not fully supported by the statistical reporting provided

Locations:

- RESULTS, L111-117 ("significantly higher... strongly correlated...")

- DISCUSSION, L175-176 ("significantly higher...")

- Figure 2

- Figure 4

- Statistical Analyses section, L521-524

Although statistical claims such as "significantly higher" and "strongly correlated" are made in the RESULTS and DISCUSSION sections, the manuscript does not provide the statistical evidence necessary to support these statements.

Issues identified:

- The MATERIALS AND METHODS do not specify which statistical tests were used for group comparisons (e.g., t-test, ANOVA, post-hoc tests).

- Figure 2 does not include significance indicators (e.g., *, **), making it impossible to verify statements of statistical significance.

- Figure 4 also lacks significance indicators, and the statistical treatment of RT-qPCR data is not described.

- No P values are reported in the text.

- Although Table S1 is cited once in the DISCUSSION, the initial correlation claims in the RESULTS (L116-117) do not reference Table S1, leaving readers unable to identify the source of the correlation analysis.

Required actions:

- Indicate the statistical tests used for metabolite comparisons and qPCR data (e.g., t-test, ANOVA with post-hoc corrections).

- Add significance markers to Figures 2 and 4 and clarify the comparisons tested.

- When correlations are first mentioned in the RESULTS, explicitly reference Table S1.

- If certain comparisons are not statistically significant, revise the text accordingly (e.g., "higher", "tended to be higher", "associated with").

5. Discussion is overly long and contains extended review-style background

Location: DISCUSSION, approximately L163-464 (entire Discussion section)

The Discussion includes extensive descriptions of prior studies across multiple plant species, often with detailed functional explanations that are not directly tied to the present results. This makes the Discussion substantially longer than necessary and dilutes the major findings of the study.

The issue is not limited to a single paragraph; the entire Discussion section would benefit from condensation and a clearer focus on:

- the key findings of the present study,

- how these findings relate to essential prior work,

- biological interpretations directly supported by the data,

- limitations and future research directions.

Reducing long summaries of gene functions in other species would greatly improve clarity and strengthen the manuscript.___________________________________

Minor Comments

m1. "Metabolomic analysis" is misleading

Location: Introduction, L82

Although the manuscript uses the term "metabolomic analysis," the measurements performed are limited to bulk quantification of major metabolite classes (TPC, TFC, TAC, TPAC, TIC), rather than profiling of individual metabolites.

To avoid overstatement, a more accurate phrase such as "metabolite measurements" or "quantification of major metabolite classes" is recommended.

m2. Inaccurate phrasing "integrating metabolite profiling and transcriptome analysis" in the Discussion

Location: DISCUSSION, L164-165

The phrase "integrating metabolite profiling and transcriptome analysis" overstates the scope of the metabolic measurements, because the study quantifies only bulk levels of major metabolite classes (TPC, TFC, TAC, TPAC, TIC) rather than profiling individual metabolites.

To maintain accuracy and avoid implying a metabolite-level survey,

a more appropriate expression would be:

- "integrating metabolite measurements with transcriptome analysis"

- "combining transcriptome data with quantification of major metabolite classes"

This wording reflects the actual analytical scope of the study more precisely.

m3. Reference typo ("Skuba" → "Tsukuba")

Location: References, L809-810

The National Food Research Institute is located in Tsukuba, Japan.

Please correct the spelling.

m4. Statistical notation formatting

Location: RESULTS, L127 etc.

In scientific writing, statistical symbols such as P, q, and Q are conventionally italicized.

Please format all statistical values accordingly.

m5. Figure 3 panel label mismatch

Location: Fig. 3 caption

Figure 3 uses (1) and (2) in the panels, but the caption uses (a) and (b).

Please align the caption with the figure.

m6. Figure 4 caption order inconsistent with figure layout

Location: Fig. 4 caption

The figure shows F3H → ANS → LAR, but the caption lists ANS first.

Please correct to match the order in the figure.

Summary

The manuscript presents valuable data and addresses an important biological question; however, several critical issues need to be resolved to meet sound-science standards. In particular, improvements are required in:

- methodological transparency, including extraction procedures, analytical conditions for HPLC and spectrophotometric/radical-scavenging assays, and clear reporting of biological replicate numbers for both metabolite assays and RT-qPCR.

- statistical reporting, such as specifying statistical tests, providing statistical P/q/Q values, adding significance indicators in Figures 2 and 4, and appropriately referencing Table S1 when discussing correlations.

- accuracy and consistency of terminology, especially regarding the description of metabolite measurements.

- organization and clarity of the Discussion, which is currently overly long and contains review-style content not directly tied to the study's findings.

Addressing these points will substantially improve the rigor, reproducibility, and readability of the manuscript

Decision Letter Round 2:

December 24, 2025

Prof. Ik-Young Choi

Kangwon National University

Chuncheon

Korea (South), Republic of

RE: Transcriptomic and Metabolic Insight into Flavonoid Biosynthesis Underlying Seed Coat Color Variation in Soybean (Glycine max)

Dear Dr. Choi:

Thank you for submitting to Plant Direct. All required reviews have been returned and we have now finished our evaluation of your manuscript. In light of the reviewers' and editor's comments, further revisions are needed before the paper can be accepted for publication in Plant Direct.

Please view the editors' and reviewers' comments below and use their suggestions as a guide while you work on your revision.

When uploading the revised version of this article, please be sure to include the following:

-A word document that contains your response to the reviewers. You should respond to each reviewer comment and note the changes made to the manuscript. If you do not agree with a reviewer's comment and choose not to make a suggested revision, please explain why. Please try to provide as complete an answer as possible to each reviewer's criticisms.

-A tracked changes document with each change highlighted

- A clean version of the latest version of the manuscript

Thank you for your carefully revised version. Both reviewers are very positive about the revisions, although some issues regarding over-interpretation of results remains. I look forward to seeing a revised version. If the comments of the reviewer are properly addressed (or refuted), I hope to be able to expedite decision without the need of another round of reviews.

To upload your revision, please click the link below.

https://plantdirect.msubmit.net/cgi-bin/main.plex?el=A2Lr2pj3B4nPI4I5A9ftd7uvTLdjPSG4Sifp6rTgFIQZ

In order to provide as timely a service as possible, we ask that your revision is resubmitted within three months after receipt of this request. If an extension is needed, please send a request, along with a brief explanation, to the editorial office at plantdirect@wiley.com .

Please note that, in addition to publishing reviewer comments, the author's responses to review comments will also be published alongside the final version of the paper. If

you would not like the author's responses to be published, please contact the editorial office at plantdirect@wiley.com .

Thank you very much for giving us an opportunity to review your work. I look forward to receiving the next version.

Sincerely,

Gustavo MacIntosh

Editor, Plant Direct

-- Editor comments:

Thank you for your carefully revised version. Both reviewers are very positive about the revisions, although some issues regarding over-interpretation of results remains. I look forward to seeing a revised version. If the comments of the reviewer are properly addressed (or refuted), I hope to be able to expedite decision without the need of another round of reviews.

-- Reviewer comments:

Reviewer #1:

Thank you so much for making the adjustment.

Reviewer #2:

Thank you for submitting the revised version of this manuscript. The authors have made substantial improvements in response to the previous review, particularly with respect to methodological transparency, statistical reporting, and clarification of experimental design. The study now largely meets Plant Direct's sound-science criteria. However, several issues related to interpretation, experimental scope, and terminological accuracy still require attention. These concerns can be addressed through clarification and careful wording, without the need for additional experiments.

Major Comments

---

1. Overstatement of "first evidence" regarding small heat shock proteins (sHSPs)

Location:

- ABSTRACT, L45-46

- DISCUSSION, Section 4.2.5 (approximately L492-499)

The manuscript states that this study provides the "first evidence" that small heat shock proteins (sHSPs) are involved in anthocyanin accumulation and seed coat coloration in soybean.

However, the evidence presented for sHSP involvement is limited to differential gene expression identified by RNA-seq at a single developmental stage. No functional validation, stress or temperature treatments, mutant analysis, or protein-level assays were performed to directly demonstrate a regulatory role of sHSPs in pigmentation.

While the observed upregulation of sHSP genes in black seed coats is interesting and may suggest an association with pigmentation or stress responses, it does not constitute functional evidence. Therefore, the claim of providing "first evidence" appears overstated.

The authors are encouraged to moderate this wording (e.g., "suggests a potential involvement" or "is associated with") and to clearly distinguish correlation from causation.

---

2. Interpretation of the LAR-PA-LAC5 relationship requires clearer qualification

Location:

- DISCUSSION, Section 4.2.2 (approximately L423-441)

The revised Discussion provides an improved and biologically plausible explanation for the apparent discrepancy between higher LAR expression in yellow seed coats and higher proanthocyanidin (PA) accumulation in black seed coats, proposing that enhanced PA polymerization mediated by LAC5 may account for this pattern.

However, no direct measurements of PA polymer size, PA solubility (soluble vs. insoluble fractions), or LAC5 enzymatic activity were performed in this study. Consequently, the proposed involvement of LAC5 in PA polymerization remains inferential and cannot be considered a demonstrated mechanism based solely on transcript abundance.

In particular, wording that implies a central or causal role of LAC5 (e.g., "plays a pivotal role in elevated PA content") exceeds the evidential scope of the presented data. The authors are encouraged to explicitly frame this interpretation as a hypothesis or working model rather than a validated mechanism.

For clarity, the authors may consider revising the relevant sentences using wording such as:

• "...suggesting that LAC5 may contribute to PA accumulation and darker pigmentation."

• "...consistent with a model in which LAC5 is potentially involved in PA polymerization during seed coat development."

• "...suggesting a possible role for LAC5 in PA accumulation, which warrants further functional validation."

Such revisions would appropriately distinguish correlation from causation and align the interpretation with the experimental evidence presented. In particular, please avoid strong causal phrasing (e.g., "plays a pivotal role") unless supported by direct evidence, and instead use wording that clearly indicates a hypothesis/model based on transcript-level association.

---

3. Limitations of single-stage RNA-seq analysis should be more explicitly discussed

Location:

- DISCUSSION, Sections 4.2.1-4.2.5 (e.g., statements referring to "molecular mechanisms," "critical regulators," or genes that "play a crucial role")

RNA-seq analysis in this study was conducted at a single developmental stage (110 DAS), when seed coat color differentiation had already become apparent. Under this experimental design, it is not possible to determine whether the observed transcriptional differences represent causal regulatory events initiating pigmentation or downstream responses associated with established color differences.

Nevertheless, the Discussion contains several statements that imply definitive regulatory or causal roles for specific genes (e.g., "elucidated the molecular mechanisms," "critical regulators," "play a crucial role"). Such wording exceeds what can be inferred from single-stage transcriptomic data. This point also applies to schematic summaries (e.g., Fig. 5), where "mechanisms" or "regulators" may be interpreted as causal without temporal or functional validation.

The authors are encouraged to more explicitly acknowledge this limitation and to moderate causal language accordingly, clarifying that the transcriptomic results reflect associations at a specific developmental stage rather than temporal regulatory hierarchies.___________________________________

Minor Comments

____

m1. Gene/protein nomenclature and homolog references remain partially inconsistent

Location:

- DISCUSSION, particularly Section 4.2.5 (approximately L483-491)

- RESULTS, Section 3.5 (approximately L347-369)

In several places, gene expression data derived from RNA-seq are discussed using protein-level terminology, and soybean genes are listed together with Arabidopsis thaliana gene names without clearly distinguishing experimentally measured genes from homolog-based functional context. This may lead to ambiguity regarding the biological level (gene vs. protein) and species context of the reported results.

Example 1 (homolog and species ambiguity):

In Discussion section 4.2.5, the manuscript states:

"The results showed the expression of GSTF11, ATGSTF6, ATGSTF11, were up regulated in the black seed soybeans."

This phrasing is ambiguous because ATGSTF6 and ATGSTF11 refer to A. thaliana genes, whereas the expression data were obtained from soybean (Glycine max).

A clearer formulation would explicitly separate soybean gene expression results from homolog-based functional references, for example:

"The soybean GSTF11 gene (Glyma.18G043700) was significantly upregulated in black seed coats. This gene is homologous to A. thaliana ATGSTF11, which has been reported to function in anthocyanin transport."

Example 2 (gene expression vs. protein function conflation):

In Results section 3.5, the manuscript states:

"Other genes specifically upregulated in black seed coats included GSTF11 (Glyma.18G043700), a glutathione S-transferase involved in anthocyanin transport to the vacuole, LAC5 (Glyma.11G233400), associated with PA polymerization, and several small heat-shock proteins (Glyma.06G134900) that may contribute to stress-responsive regulation of pigmentation."

Although RNA-seq data demonstrate upregulation of these genes, the phrasing implies protein-level functional activity within the present experimental system. To maintain accuracy, gene expression results should be clearly distinguished from protein functions inferred from previous studies. For example, this sentence could be revised as:

"Other genes specifically upregulated in black seed coats included GSTF11 (Glyma.18G043700), LAC5 (Glyma.11G233400), and several small heat-shock protein genes (e.g., Glyma.06G134900). These genes encode proteins that have been reported to function in anthocyanin transport, PA polymerization, and stress-responsive regulation, respectively."

Consistent application of this distinction throughout the manuscript would improve terminological precision and prevent confusion regarding the interpretation of transcriptomic results.

---

m2. Formatting inconsistencies in enzyme nomenclature (italicization of O and S)

Location:

- DISCUSSION, Section 4.2.5: L471, L473, L478

- ABSTRACT: L36

- RESULTS, Section 3.5: L347

- DISCUSSION: L481

The enzyme names 3-O-glucosyltransferase and glutathione S-transferase appear with inconsistent formatting, particularly with respect to the italicization of the letters "O" and "S."

As these letters are integral components of standard biochemical enzyme nomenclature, consistent italicization throughout the manuscript is recommended to conform with established biochemical and molecular biology conventions.

m3. Minor clarity and presentation issues

Location:

- DISCUSSION and Figure 5

A final careful check is recommended to improve clarity and consistency in presentation.

First, although major instances of gene/protein nomenclature inconsistency have been addressed above, minor cases remain in the Discussion where gene expression results and protein-level functional descriptions are interwoven within the same sentences or paragraphs. Further standardization would improve readability and precision.

Second, in Figure 5, transcription factors such as MYB17 are presented alongside well-characterized regulators (e.g., MYB113), which may give the impression of equivalent regulatory status. Given that the current study reports differential expression but does not provide functional validation for MYB17, clearer labeling or a more cautious interpretation in the figure legend would help align the schematic representation with the strength of the experimental evidence.

Finally, although the Discussion has been substantially condensed, some review-style descriptions remain, particularly in the sHSP and CYP-related sections. Further focusing these paragraphs on the study's own results, while minimizing extended background from other species, would strengthen the data-centered narrative and improve overall conciseness.

Summary

The revised manuscript represents a clear improvement over the previous version and largely satisfies sound-science requirements. The remaining issues primarily concern interpretational scope, wording precision, and terminological consistency, rather than data quality or experimental rigor.

Addressing the points above-particularly moderation of overstatements and clarification of limitations-will further strengthen the manuscript and improve its clarity and accuracy for readers.

Decision Letter Round 3:

February 3, 2026

Prof. Ik-Young Choi

Kangwon National University

Chuncheon

Korea (South), Republic of

MSID: 2025-01842-TWR2

MS TITLE: Transcriptomic and Metabolic Insight into Flavonoid Biosynthesis Underlying Black and Yellow Seed Coat Color Variation in Soybean (Glycine max)

Dear Dr. Ik-Young Choi:

I am pleased to inform you that your manuscript "Transcriptomic and Metabolic Insight into Flavonoid Biosynthesis Underlying Black and Yellow Seed Coat Color Variation in Soybean (Glycine max)" has been accepted for publication in Plant Direct.

Your article will appear online in the next available issue of Plant Direct. To ensure your article gets published as quickly as possible, please pay attention to the steps detailed below. We have found that most of the delays happen at this stage, especially at the payment stage, so please respond as quickly as possible when prompted.

License Agreement: Once your article has been accepted it will move to Production and undergo admin and file checking - you may receive an email with any queries we have at this stage. When all required items are received by the publisher and queries resolved, the corresponding author will receive an email from Wiley's Author Services system which will ask them to log on at https://authorservices.wiley.com/bauthor and will present them with the appropriate license for completion. Your article cannot be published until both the signed license agreement and payment of the article fee have been received.

Payment of the Open Access Article Publication Fee: All articles published in Plant Direct are fully open access: immediately and freely available to read, download and share. Plant Direct charges a publication fee to cover the publication costs. The corresponding author for this manuscript should have already received a quote from the payments team (cs-openaccess@wiley.com) with the estimated article publication fee; please email cs-openaccess@wiley.com if this has not been received. The corresponding author should log on to the Wiley Author Services site, where the publication fee can be paid by credit card or an invoice. Pro Forma can also be requested. Payment of the publication charge must be received before the article will be published online.

Proofs: You will have the opportunity to look over your paper once more when you receive the author proofs for your article. The proofs will be with you in approximately two weeks. Please note that, in addition to publishing reviewer comments, the author's responses to review comments will also be published alongside the final version of the paper. If you would not like the author's responses to be published, please contact the editorial office at plantdirect@wiley.com .

Promotion of your article: You can help your research get the attention it deserves! Check out Wiley's free Promotion Guide for best-practice recommendations for promoting your work at www.wileyauthors.com/eeo/guide. And learn more about Wiley Editing Services which offers professional video, design, and writing services to create shareable video abstracts, infographics, conference posters, lay summaries, and research news stories for your research at www.wileyauthors.com/eeo/promotion.

Thank you again for your contribution to Plant Direct. If you have any questions, feel free to contact the editorial office at plantdirect@wiley.com .

Sincerely,

Gustavo MacIntosh

Gustavo MacIntosh

Editor, Plant Direct

Response Letter Round 1:

Reviewer 1:

The manuscript presents a comprehensive transcriptomic and metabolomic analysis to investigate the genetic basis of flavonoid biosynthesis underlying seed coat color variation in soybean. The study is well-conceived and demonstrates a significant experimental effort, particularly in developing recombinant inbred lines (RILs) and integrating multiple data types. However, while the overall framework is strong, several aspects require substantial improvement before the work can be considered for publication. These include clarity in data presentation, justification of methodological choices, inclusion of missing experimental details, and integration of relevant literature to position this work within the broader context. Therefore, I recommend some revisions for the manuscript.

Major Comments

1. The manuscript demonstrates commendable effort in generating RIL lines and performing RNA-seq and metabolite analyses. However, the novelty and positioning of the study within existing literature need clearer articulation. The introduction should highlight prior transcriptomic and metabolic studies on similar traits to better define the research gap.

Response:

Thank you for your insightful feedback, which has greatly improved our manuscript. We have substantially revised the Introduction (Line number 84-96) to highlight prior studies. We believe these additions explicitly define the research gap.

2. The choice of reference genome (Wm82.a2.v1) for transcriptomic analysis requires justification, especially since newer annotations (e.g., W82.a6) are available. The authors should

discuss why this specific version was chosen and how it affects the interpretation of results.

Response:

We sincerely appreciate your valuable comment. We are sorry for our careless mistake. In

previous submitted manuscript we used Wm82.a4.v1 as a reference genome. The reference

genome Wm82.a4.v1has been consistently used in our previous studies. Moreover, this version

(Wm82.a4.v1) was available and integrated into our institutional bioinformatics pipeline, which

helped efficient data processing and analysis. Since the data for this study were generated and

analyzed in 2023, we considered Wm82.a4.v1 appropriate at that time. We believe the core gene

models and genomic structure using Wm82.a4.v1 remain conserved and we do not expect the

choice of Wm82.a4.v1 alter the biological interpretation of our results. Nevertheless, we

recognize the advantages of newer annotations and plan to incorporate the updated reference

W82.a6 genome in our future research to further enhance accuracy and interpretation.

3. The functional annotation section (Lines 142–153) should clearly indicate characterized gene

functions such as CYP73A5 (C4H) and CYP82C4 and others. Accurate functional assignment is

crucial for interpreting the biological relevance of the DEGs.

Response:

Thank you for your comment. In the revised Result section (Line number: 343-370) we

characterized the functions for the major genes such as CYP73A5 (C4H) and CYP82C4 and

others with references. We believe these improvements will strengthen the biological

interpretation of the DEG patterns in relation to seed coat pigmentation.

4. Gene Ontology (GO) and KEGG enrichment analyses should be presented before the

discussion section and supported by a suitable visual representation such as a bubble or bar plot.

A volcano plot summarizing the DEGs would also greatly improve data visualization.

Response:

Thank you for your comment. In the revised manuscript, we have moved the Gene Ontology

(GO) and KEGG enrichment analysis as a subsection in the Results section placed before the

Discussion (Line number: 320-342). We now present these data with clearly labeled bar plots to

facilitate interpretation. We are extremely sorry we didn’t generate the volcano plot for these

DEGs.

5. Table 1 only lists two samples. Clarification is needed regarding biological replicates. Were

replicates included in the RNA-seq experiment? Without this information, statistical robustness

and reproducibility cannot be verified.

Response:

Thank you for pointing out regarding biological replication. In this study, RNA-seq was

performed using three independent biological replicates for each seed coat color (Yellow and

Black). The summary values shown in Table 1 represent the mean read statistics across the three

replicates per group. For more information we have clarified the number of replicates of samples

in the Materials and Methods section. (Line Number – 190-192)

6. The Materials and Methods section lacks critical details necessary for reproducibility. The

authors should include all software tools, R packages, and parameters used for transcriptomic

and enrichment analyses, ideally in supplementary materials.

Response:

Thank you for emphasizing the importance of methodological transparency and reproducibility.

In the revised Manuscript (Materials and Methods) we have added detailed information in all

bioinformatic steps including software used for quality control, trimming, alignment, read count

and DEGs analysis (Line number: 189-236).

7. The authors should justify why metabolite profiling was performed only via HPLC and not

mass spectrometry (MS), which could have provided broader metabolite coverage and increased

resolution.

Response:

Thank you for comment regarding the choice of analytical platform. In current study we target

seed coat metabolites such as isoflavones, proanthocyanidins, anthocyanins, and total

phenolics/flavonoids. We trust, for these compounds, HPLC would be the best platform and is

widely used for soybean seed coat phenotyping. Due to limited instrument access and project

budget, implementing LC-MS was not possible within current study. However, we agree that LCMS would enable broader coverage and could reveal additional pigmentation related feature. We

will present LC-MS as an important direction for upcoming future research projects.

8. The manuscript should include the NCBI BioProject or SRA accession numbers for the RNAseq data to ensure transparency and reproducibility.

Response:

Thank you for highlighting this important point. The raw and processed RNA-seq data have now

been submitted to the NCBI GEO repository. The raw sequencing data were deposited into the

NCBI SRA database with accession number GSE313134 (Line number – 197 to 198).

9. The qPCR validation section should include information about the number of biological and

technical replicates, primer efficiencies, and controls used to validate the RNA-seq data.

Response:

Thank you for this helpful comment. We are sorry we didn't include these in Manuscript. In the

revised version we have added the information about the number of biological and technical

repeats and controls used to validate the RNA-seq data (Line number: 224-236).

10. The enrichment analysis currently appears to use a generalized method. The authors are

encouraged to employ Gene Set Enrichment Analysis (GSEA), which provides a more robust and

biologically meaningful interpretation of DEGs.

Response:

Thank you for valuable suggestions. In the present study, enrichment was performed using

significantly differentially expressed genes, with the aim focusing on pathways showing

transcriptional changes. We are very sorry, we did not apply Gene Set Enrichment Analysis

(GSEA) and re-analyzing all the datasets with GSEA is beyond the scope of the current study.

We have added a note that in coming research we will be using GSEA as an important direction

for data analysis.

Minor Comments

1. All scientific names (e.g., Glycine max, Glycine soja) should be italicized throughout the text,

including figure captions and tables.

Response:

Thank you for pointing this out. In the revised manuscript, all scientific names of species and subspecies (e.g. Glycine max, Glycine soja) have been consistently italicized throughout the main text, figure captions, and tables.

2. Line 55: Include recent quantitative data on the average oil and protein percentage in soybean seeds to improve the contextual depth of the introduction.

Response:

We appreciate your suggestion. In the revised introduction (Line number-56 to 57), we included information on soybean seed composition based on recent statistics and compositional reviews.

3. The manuscript would benefit from careful language polishing to correct grammatical inconsistencies and typographical errors.

Response:

We appreciated your observation. The revised manuscript has now been carefully edited to correct grammatical inconsistencies and typographical errors.

4. Figure 4: Please specify how many biological replicates were used for RT-qPCR validation.

Response:

Thank you for your comment. For the validation of RT-qPCR three technical replicates were conducted for each sample (Line number 234 -236).

5. The figure legends should be more descriptive, including sample type, statistical tests, and pvalues where relevant.

Response:

Thank you for your comment. In the revised manuscript, all figure legends have been clearly

specified the sample types and groups, the number of biological replicates, and the statistical

tests applied, including significance thresholds and p-values where relevant.

6. The authors should ensure consistent gene name formatting (italicize gene symbols, regular

font for proteins).

Response:

Thank you for this helpful suggestion. In the revised manuscript, we have standardized our gene

symbols are consistently written in italics, whereas the corresponding proteins are presented in

regular font.

Reviewer #2:

Review Comments to the Authors (with line numbers)

Thank you for submitting this manuscript. The topic is relevant and the dataset has potential

value; However, several essential issues regarding methodological transparency, statistical

reporting, and clarity of interpretation need to be addressed to ensure reproducibility and

alignment with Plant Direct's sound-science criteria. Below are detailed comments.

Major Comments

1. Biological replicate number (n) missing for metabolite assays and RT-qPCR assays

Location:

- RESULTS, L111-117

- Figure 2 legend

- Figure 4 legend

- MATERIALS AND METHODS, Metabolite Analysis & RT-qPCR sections

Figure 2 reports mean {plus minus} SD values, but the number of biological replicates (n) is not

stated in the figure, its legend, or the MATERIALS AND METHODS.

Without n, the robustness and reproducibility of the metabolite data cannot be evaluated.

Please specify the biological replicate number for each measurement, and consider providing

replicate-level data in supplementary materials.

In addition to Fig. 2, Fig. 4 also lacks information on the number of biological replicates (n).

Even though error bars are shown (standard error), the absence of n makes it impossible to assess

the robustness of the RT-qPCR validation.

Please specify the number of biological replicates used for each qPCR assay.

Response:

Thank you for your kind comments. In the revised manuscript, we now specify the number of

replications for all experiments. In the Materials and Methods, we state that seed coat color and

metabolite measurements were obtained from three independent biological replicates per line

(Line number 131), each analyzed with three technical replicates, and that RT-qPCR assays were

performed with the same number of biological and technical replicates (Line number 234, 235).

We also indicate that data are presented as mean ± standard deviation of three biological

replicates and that statistical significance between black and yellow lines was assessed using

Student's t-test ($p < 0.05$). Also, figure legends have been updated accordingly.

2. Sample extraction and preparation methods insufficiently described

Location: Metabolite Analysis section, L493-498

Although several published protocols are cited, the manuscript does not describe:

- seed grinding method,

- extraction solvent(s), ratio, or volume,

- extraction temperature, time, or equipment,

- centrifugation/filtration steps.

These steps are essential for reproducibility, even when established methods are cited.

Please include a concise but complete description of extraction procedures.

Response:

Thank you for pointing out this important issue. In the revised Metabolite Analysis (Materials

and methods) section, we have now provided a concise and complete description of the

extraction procedures. Specifically, we describe the seed grinding method, the extraction solvent

and solvent to sample ratio, extraction volume, temperature, duration, and equipment used as

well as the subsequent centrifugation and syringe-filtration steps prior to HPLC or

spectrophotometric analysis (Line number -128 to 188).

3. Analytical conditions for HPLC, spectrophotometry, and radical scavenging assays are

insufficiently described

Location: Metabolite Analysis section, L493-498

The manuscript does not provide the essential analytical conditions required for reproducing any

of the metabolite assays. Missing information includes:

- Instrument information (manufacturer/model of HPLC system, spectrophotometer/microplate

reader, etc.)

- HPLC conditions (column type/dimensions, mobile phase, gradient, flow rate, wavelength,

temperature)

- Spectrophotometric assay conditions (wavelengths, incubation times, standard curves,

calculation methods)

- DPPH/ABTS assay conditions (reagent concentrations, reaction times, wavelengths, %

inhibition formula)

Because these details are absent, the assays cannot be reproduced.

Please provide the minimum essential analytical conditions to ensure reproducibility.

Response:

Thank you for your comment. In the revised Metabolite Analysis section, we have added the

essential analytical conditions for all assays. HPLC conditions (column type and dimensions,

mobile phase composition, gradient program, flow rate, column temperature, and detection

wavelength). In addition, we describe the DPPH and ABTS assays in full, including reagent

concentrations, reaction times, measurement wavelengths, and the equations used to calculate

radical scavenging (% inhibition) (Line number – 128 to 188).

4. Statistical claims ("significantly higher", "strongly correlated") are not fully supported by the

statistical reporting provided

Locations:

- RESULTS, L111-117 ("significantly higher... strongly correlated...")

- DISCUSSION, L175-176 ("significantly higher...")

- Figure 2

- Figure 4

- Statistical Analyses section, L521-524

Although statistical claims such as "significantly higher" and "strongly correlated" are made in

the RESULTS and DISCUSSION sections, the manuscript does not provide the statistical

evidence necessary to support these statements.

Issues identified:

- The MATERIALS AND METHODS do not specify which statistical tests were used for group

comparisons (eg, t-test, ANOVA, post-hoc tests).

- Figure 2 does not include significance indicators (eg, *, **), making it impossible to verify

statements of statistical significance.

- Figure 4 also lacks significance indicators, and the statistical treatment of RT-qPCR data is not

described.

- No P values are reported in the text.

- Although Table S1 is cited once in the DISCUSSION, the initial correlation claims in the

RESULTS (L116-117) do not reference Table S1, leaving readers unable to identify the source of

the correlation analysis.

Required actions:

- Indicate the statistical tests used for metabolite comparisons and qPCR data (eg, t-test, ANOVA

with post-hoc corrections).

- Add significance markers to Figures 2 and 4 and clarify the comparisons tested.

- When correlations are first mentioned in the RESULTS, explicitly reference Table S1.

- If certain comparisons are not statistically significant, revise the text accordingly (eg, "higher",

"tended to be higher", "associated with").

Response:

Thank you for these careful statistical suggestions. In the revised manuscript, we now (i) specify

in the Statistical Analyses section the tests used for group comparisons of metabolite and

RT-qPCR data (Student's t-test, $p < 0.05$), (ii) add significance indicators (*) to Figures 2 and 4

and clearly state in the legends which pairwise comparisons were tested, (iii) report p-values or

significance levels in the text where "significantly higher" is claimed, and (iv) cite Table S1 at

the first mention of correlation analyses in the Results (Line number- 298 to 307).

5. Discussion is overly long and contains extended review-style background

Location: DISCUSSION, approximately L163-464 (entire Discussion section)

The Discussion includes extensive descriptions of prior studies across multiple plant species,

often with detailed functional explanations that are not directly tied to the present results. This

makes the discussion substantially longer than necessary and dilutes the major findings of the

study.

The issue is not limited to a single paragraph; the entire Discussion section would benefit from

condensation and a clearer focus on:

- the key findings of the present study,

- how these findings relate to essential prior work,

- biological interpretations directly supported by the data,

- limitations and future research directions.

Reducing long summaries of gene functions in other species would greatly improve clarity and

strengthen the manuscript.

Response:

Thank you for the constructive comment on the Discussion section. The Discussion has now

been substantially revised to address these concerns. Specifically, the length has been reduced by

removing extended, review-style background and detailed descriptions of gene functions. The

revised Discussion is now well structured according to given suggestions. Also, long summaries

of previous studies across multiple plant species have been deleted with the core results to

improve the clarity and focus on the main contributions of the manuscript.

Minor Comments

m1. "Metabolomic analysis" is misleading

Location: Introduction, L82

Although the manuscript uses the term "metabolomic analysis," the measurements performed are

limited to bulk quantification of major metabolite classes (TPC, TFC, TAC, TPAC, TIC), rather

than profiling of individual metabolites.

To avoid overstatement, a more accurate phrase such as “metabolite measurements” or

“quantification of major metabolite classes” is recommended.

Response:

Thank you for the comments. In the revised manuscript in Introduction section, we have

corrected the phrase and now reads “major metabolite measurement”. We agreed this gives more

accurate description (Line number - 98).

m2. Inaccurate phrasing "integrating metabolite profiling and transcriptome analysis" in the

Discussion

Location: DISCUSSION, L164-165

The phrase "integrating metabolite profiling and transcriptome analysis" overstates the scope of

the metabolic measurements, because the study quantifies only bulk levels of major metabolite

classes (TPC, TFC, TAC, TPAC, TIC) rather than profiling individual metabolites.

To maintain accuracy and avoid implying a metabolite-level survey,

a more appropriate expression would be:

- "integrating metabolite measurements with transcriptome analysis"
- "combining transcriptome data with quantification of major metabolite classes"

This wording reflects the actual analytical scope of the study more precisely.

Response:

Thank you for your insightful comments. In the revised manuscript in Discussion section, we

have corrected the phrase now reads, “by integrating metabolite measurements with

transcriptome analysis”. (Line number - 384)

m3. Reference typo ("Skuba" → "Tsukuba")

Location: References, L809-810

The National Food Research Institute is located in Tsukuba, Japan.

Please correct the spelling.

Response:

We are sorry for our careless mistake. Now in the revised manuscript we have corrected the

spelling (Line number- 699).

m4. Statistical notation formatting

Location: RESULTS, L127 etc.

In scientific writing, statistical symbols such as P, q, and Q are conventionally italicized.

Please format all statistical values accordingly.

Response:

We are sorry for our careless mistake. All statistical symbols, including P, q, and Q, have been

reformatted to italic font throughout the Results section and also the entire manuscript.

m5. Figure 3 panel label mismatch

Location: Fig. 3 caption

Figure 3 uses (1) and (2) in the panels, but the caption uses (a) and (b).

Please align the caption with the figure.

Response:

We are sorry for our careless mistake. We have corrected the caption of Figure 3 (Revised

manuscript – Figure 2) to match the panel labels used in the figure with (a) and (b).

m6. Figure 4 caption order inconsistent with figure layout

Location: Fig. 4 caption

The figure shows F3H → ANS → LAR, but the caption lists ANS first.

Please correct to match the order in the figure.

Response:

Thank you for identifying this inconsistency. The caption of Figure 4 has been revised to reflect

the correct order of enzymes as presented in the figure (F3H-3 → ANS → LAR).

Summary

The manuscript presents valuable data and addresses an important biological question; However,

several critical issues need to be resolved to meet sound-science standards. In particular,

improvements are required in:

- methodological transparency, including extraction procedures, analytical conditions for HPLC

and spectrophotometric/radical-scavenging assays, and clear reporting of biological replicate

numbers for both metabolite assays and RT-qPCR.

- statistical reporting, such as specifying statistical tests, providing statistical P/q/Q values,

adding significance indicators in Figures 2 and 4, and appropriately referencing Table S1 when

discussing correlations.

- Accuracy and consistency of terminology, especially regarding the description of metabolite

measurements.

- organization and clarity of the Discussion, which is currently overly long and contains reviewstyle content not directly tied to the study's findings.

Addressing these points will substantially improve the rigor, reproducibility, and readability of

the manuscript.

Thank you for the comprehensive and constructive feedback. All specified issues have been

thoroughly addressed in the revised manuscript. Materials and methods section – with details

extraction protocols, HPLC conditions, biological replicates and statistical analysis. The

discussion part has been shortened on key study findings. We believe these changes and revised

manuscript will enhance rigor, reproducibility, and readability of the manuscript.

Response Letter Round 2:

Reviewer 1:

The manuscript presents a comprehensive transcriptomic and metabolomic analysis to

investigate the genetic basis of flavonoid biosynthesis underlying seed coat color variation in

soybean. The study is well-conceived and demonstrates a significant experimental effort,

particularly in developing recombinant inbred lines (RILs) and integrating multiple data types.

However, while the overall framework is strong, several aspects require substantial improvement

before the work can be considered for publication. These include clarity in data presentation,

justification of methodological choices, inclusion of missing experimental details, and

integration of relevant literature to position this work within the broader context. Therefore, I

recommend some revisions for the manuscript.

Major Comments

1. The manuscript demonstrates commendable effort in generating RIL lines and performing

RNA-seq and metabolite analyses. However, the novelty and positioning of the study within

existing literature need clearer articulation. The introduction should highlight prior

transcriptomic and metabolic studies on similar traits to better define the research gap.

Response:

Thank you for your insightful feedback, which has greatly improved our manuscript. We have

substantially revised the Introduction (Line number 84-96) to highlight prior studies. We believe

these additions explicitly define the research gap.

2. The choice of reference genome (Wm82.a2.v1) for transcriptomic analysis requires

justification, especially since newer annotations (e.g., W82.a6) are available. The authors should

discuss why this specific version was chosen and how it affects the interpretation of results.

Response:

We sincerely appreciate your valuable comment. We are sorry for our careless mistake. In

previous submitted manuscript we used Wm82.a4.v1 as a reference genome. The reference

genome Wm82.a4.v1has been consistently used in our previous studies. Moreover, this version

(Wm82.a4.v1) was available and integrated into our institutional bioinformatics pipeline, which

helped efficient data processing and analysis. Since the data for this study were generated and

analyzed in 2023, we considered Wm82.a4.v1 appropriate at that time. We believe the core gene

models and genomic structure using Wm82.a4.v1 remain conserved and we do not expect the

choice of Wm82.a4.v1 alter the biological interpretation of our results. Nevertheless, we

recognize the advantages of newer annotations and plan to incorporate the updated reference

W82.a6 genome in our future research to further enhance accuracy and interpretation.

3. The functional annotation section (Lines 142–153) should clearly indicate characterized gene

functions such as CYP73A5 (C4H) and CYP82C4 and others. Accurate functional assignment is

crucial for interpreting the biological relevance of the DEGs.

Response:

Thank you for your comment. In the revised Result section (Line number: 343-370) we

characterized the functions for the major genes such as CYP73A5 (C4H) and CYP82C4 and

others with references. We believe these improvements will strengthen the biological

interpretation of the DEG patterns in relation to seed coat pigmentation.

4. Gene Ontology (GO) and KEGG enrichment analyses should be presented before the

discussion section and supported by a suitable visual representation such as a bubble or bar plot.

A volcano plot summarizing the DEGs would also greatly improve data visualization.

Response:

Thank you for your comment. In the revised manuscript, we have moved the Gene Ontology (GO) and KEGG enrichment analysis as a subsection in the Results section placed before the Discussion (Line number: 320-342). We now present these data with clearly labeled bar plots to facilitate interpretation. We are extremely sorry we didn't generate the volcano plot for these DEGs.

5. Table 1 only lists two samples. Clarification is needed regarding biological replicates. Were replicates included in the RNA-seq experiment? Without this information, statistical robustness and reproducibility cannot be verified.

Response:

Thank you for pointing out regarding biological replication. In this study, RNA-seq was performed using three independent biological replicates for each seed coat color (Yellow and Black). The summary values shown in Table 1 represent the mean read statistics across the three replicates per group. For more information we have clarified the number of replicates of samples in the Materials and Methods section. (Line Number – 190-192)

6. The Materials and Methods section lacks critical details necessary for reproducibility. The authors should include all software tools, R packages, and parameters used for transcriptomic and enrichment analyses, ideally in supplementary materials.

Response:

Thank you for emphasizing the importance of methodological transparency and reproducibility.

In the revised Manuscript (Materials and Methods) we have added detailed information in all

bioinformatic steps including software used for quality control, trimming, alignment, read count

and DEGs analysis (Line number: 189-236).

7. The authors should justify why metabolite profiling was performed only via HPLC and not

mass spectrometry (MS), which could have provided broader metabolite coverage and increased

resolution.

Response:

Thank you for comment regarding the choice of analytical platform. In current study we target

seed coat metabolites such as isoflavones, proanthocyanidins, anthocyanins, and total

phenolics/flavonoids. We trust, for these compounds, HPLC would be the best platform and is

widely used for soybean seed coat phenotyping. Due to limited instrument access and project

budget, implementing LC-MS was not possible within current study. However, we agree that LCMS would enable broader coverage and could reveal additional pigmentation related feature. We

will present LC-MS as an important direction for upcoming future research projects.

8. The manuscript should include the NCBI BioProject or SRA accession numbers for the RNAseq data to ensure transparency and reproducibility.

Response:

Thank you for highlighting this important point. The raw and processed RNA-seq data have now

been submitted to the NCBI GEO repository. The raw sequencing data were deposited into the

NCBI SRA database with accession number GSE313134 (Line number – 197 to 198).

9. The qPCR validation section should include information about the number of biological and

technical replicates, primer efficiencies, and controls used to validate the RNA-seq data.

Response:

Thank you for this helpful comment. We are sorry we didn't include these in Manuscript. In the

revised version we have added the information about the number of biological and technical

repeats and controls used to validate the RNA-seq data (Line number: 224-236).

10. The enrichment analysis currently appears to use a generalized method. The authors are

encouraged to employ Gene Set Enrichment Analysis (GSEA), which provides a more robust and

biologically meaningful interpretation of DEGs.

Response:

Thank you for valuable suggestions. In the present study, enrichment was performed using

significantly differentially expressed genes, with the aim focusing on pathways showing

transcriptional changes. We are very sorry, we did not apply Gene Set Enrichment Analysis

(GSEA) and re-analyzing all the datasets with GSEA is beyond the scope of the current study.

We have added a note that in coming research we will be using GSEA as an important direction

for data analysis.

Minor Comments

1. All scientific names (e.g., Glycine max, Glycine soja) should be italicized throughout the text,

including figure captions and tables.

Response:

Thank you for pointing this out. In the revised manuscript, all scientific names of species and

subspecies (e.g. Glycine max, Glycine soja) have been consistently italicized throughout the

main text, figure captions, and tables.

2. Line 55: Include recent quantitative data on the average oil and protein percentage in soybean

seeds to improve the contextual depth of the introduction.

Response:

We appreciate your suggestion. In the revised introduction (Line number-56 to 57), we included

information on soybean seed composition based on recent statistics and compositional reviews.

3. The manuscript would benefit from careful language polishing to correct grammatical

inconsistencies and typographical errors.

Response:

We appreciated your observation. The revised manuscript has now been carefully edited to

correct grammatical inconsistencies and typographical errors.

4. Figure 4: Please specify how many biological replicates were used for RT-qPCR validation.

Response:

Thank you for your comment. For the validation of RT-qPCR three technical replicates were

conducted for each sample (Line number 234 -236).

5. The figure legends should be more descriptive, including sample type, statistical tests, and pvalues where relevant.

Response:

Thank you for your comment. In the revised manuscript, all figure legends have been clearly

specified the sample types and groups, the number of biological replicates, and the statistical

tests applied, including significance thresholds and p-values where relevant.

6. The authors should ensure consistent gene name formatting (italicize gene symbols, regular

font for proteins).

Response:

Thank you for this helpful suggestion. In the revised manuscript, we have standardized our gene

symbols are consistently written in italics, whereas the corresponding proteins are presented in

regular font.

Reviewer #2:

Review Comments to the Authors (with line numbers)

Thank you for submitting this manuscript. The topic is relevant and the dataset has potential

value; However, several essential issues regarding methodological transparency, statistical

reporting, and clarity of interpretation need to be addressed to ensure reproducibility and

alignment with Plant Direct's sound-science criteria. Below are detailed comments.

Major Comments

1. Biological replicate number (n) missing for metabolite assays and RT-qPCR assays

Location:

- RESULTS, L111-117

- Figure 2 legend

- Figure 4 legend

- MATERIALS AND METHODS, Metabolite Analysis & RT-qPCR sections

Figure 2 reports mean {plus minus} SD values, but the number of biological replicates (n) is not

stated in the figure, its legend, or the MATERIALS AND METHODS.

Without n, the robustness and reproducibility of the metabolite data cannot be evaluated.

Please specify the biological replicate number for each measurement, and consider providing

replicate-level data in supplementary materials.

In addition to Fig. 2, Fig. 4 also lacks information on the number of biological replicates (n).

Even though error bars are shown (standard error), the absence of n makes it impossible to assess

the robustness of the RT-qPCR validation.

Please specify the number of biological replicates used for each qPCR assay.

Response:

Thank you for your kind comments. In the revised manuscript, we now specify the number of

replications for all experiments. In the Materials and Methods, we state that seed coat color and

metabolite measurements were obtained from three independent biological replicates per line

(Line number 131), each analyzed with three technical replicates, and that RT-qPCR assays were

performed with the same number of biological and technical replicates (Line number 234, 235).

We also indicate that data are presented as mean ± standard deviation of three biological

replicates and that statistical significance between black and yellow lines was assessed using

Student's t-test ($p < 0.05$). Also, figure legends have been updated accordingly.

2. Sample extraction and preparation methods insufficiently described

Location: Metabolite Analysis section, L493-498

Although several published protocols are cited, the manuscript does not describe:

- seed grinding method,

- extraction solvent(s), ratio, or volume,

- extraction temperature, time, or equipment,

- centrifugation/filtration steps.

These steps are essential for reproducibility, even when established methods are cited.

Please include a concise but complete description of extraction procedures.

Response:

Thank you for pointing out this important issue. In the revised Metabolite Analysis (Materials

and methods) section, we have now provided a concise and complete description of the

extraction procedures. Specifically, we describe the seed grinding method, the extraction solvent

and solvent to sample ratio, extraction volume, temperature, duration, and equipment used as

well as the subsequent centrifugation and syringe-filtration steps prior to HPLC or

spectrophotometric analysis (Line number -128 to 188).

3. Analytical conditions for HPLC, spectrophotometry, and radical scavenging assays are

insufficiently described

Location: Metabolite Analysis section, L493-498

The manuscript does not provide the essential analytical conditions required for reproducing any

of the metabolite assays. Missing information includes:

- Instrument information (manufacturer/model of HPLC system, spectrophotometer/microplate

reader, etc.)

- HPLC conditions (column type/dimensions, mobile phase, gradient, flow rate, wavelength,

temperature)

- Spectrophotometric assay conditions (wavelengths, incubation times, standard curves,

calculation methods)

- DPPH/ABTS assay conditions (reagent concentrations, reaction times, wavelengths, %

inhibition formula)

Because these details are absent, the assays cannot be reproduced.

Please provide the minimum essential analytical conditions to ensure reproducibility.

Response:

Thank you for your comment. In the revised Metabolite Analysis section, we have added the

essential analytical conditions for all assays. HPLC conditions (column type and dimensions,

mobile phase composition, gradient program, flow rate, column temperature, and detection

wavelength). In addition, we describe the DPPH and ABTS assays in full, including reagent

concentrations, reaction times, measurement wavelengths, and the equations used to calculate

radical scavenging (% inhibition) (Line number – 128 to 188).

4. Statistical claims ("significantly higher", "strongly correlated") are not fully supported by the

statistical reporting provided

Locations:

- RESULTS, L111-117 ("significantly higher... strongly correlated...")

- DISCUSSION, L175-176 ("significantly higher...")

- Figure 2

- Figure 4

- Statistical Analyses section, L521-524

Although statistical claims such as "significantly higher" and "strongly correlated" are made in

the RESULTS and DISCUSSION sections, the manuscript does not provide the statistical

evidence necessary to support these statements.

Issues identified:

- The MATERIALS AND METHODS do not specify which statistical tests were used for group

comparisons (eg, t-test, ANOVA, post-hoc tests).

- Figure 2 does not include significance indicators (eg, *, **), making it impossible to verify

statements of statistical significance.

- Figure 4 also lacks significance indicators, and the statistical treatment of RT-qPCR data is not

described.

- No P values are reported in the text.

- Although Table S1 is cited once in the DISCUSSION, the initial correlation claims in the

RESULTS (L116-117) do not reference Table S1, leaving readers unable to identify the source of

the correlation analysis.

Required actions:

- Indicate the statistical tests used for metabolite comparisons and qPCR data (eg, t-test, ANOVA

with post-hoc corrections).

- Add significance markers to Figures 2 and 4 and clarify the comparisons tested.

- When correlations are first mentioned in the RESULTS, explicitly reference Table S1.

- If certain comparisons are not statistically significant, revise the text accordingly (eg, "higher",

"tended to be higher", "associated with").

Response:

Thank you for these careful statistical suggestions. In the revised manuscript, we now (i) specify

in the Statistical Analyses section the tests used for group comparisons of metabolite and

RT-qPCR data (Student’s t-test, $p < 0.05$), (ii) add significance indicators (*) to Figures 2 and 4

and clearly state in the legends which pairwise comparisons were tested, (iii) report p-values or

significance levels in the text where “significantly higher” is claimed, and (iv) cite Table S1 at

the first mention of correlation analyses in the Results (Line number- 298 to 307).

5. Discussion is overly long and contains extended review-style background

Location: DISCUSSION, approximately L163-464 (entire Discussion section)

The Discussion includes extensive descriptions of prior studies across multiple plant species,

often with detailed functional explanations that are not directly tied to the present results. This

makes the discussion substantially longer than necessary and dilutes the major findings of the

study.

The issue is not limited to a single paragraph; the entire Discussion section would benefit from

condensation and a clearer focus on:

- the key findings of the present study,
- how these findings relate to essential prior work,
- biological interpretations directly supported by the data,
- limitations and future research directions.

Reducing long summaries of gene functions in other species would greatly improve clarity and

strengthen the manuscript.

Response:

Thank you for the constructive comment on the Discussion section. The Discussion has now

been substantially revised to address these concerns. Specifically, the length has been reduced by

removing extended, review-style background and detailed descriptions of gene functions. The

revised Discussion is now well structured according to given suggestions. Also, long summaries

of previous studies across multiple plant species have been deleted with the core results to

improve the clarity and focus on the main contributions of the manuscript.

Minor Comments

m1. "Metabolomic analysis" is misleading

Location: Introduction, L82

Although the manuscript uses the term "metabolomic analysis," the measurements performed are

limited to bulk quantification of major metabolite classes (TPC, TFC, TAC, TPAC, TIC), rather

than profiling of individual metabolites.

To avoid overstatement, a more accurate phrase such as “metabolite measurements” or

“quantification of major metabolite classes” is recommended.

Response:

Thank you for the comments. In the revised manuscript in Introduction section, we have

corrected the phrase and now reads “major metabolite measurement”. We agreed this gives more

accurate description (Line number - 98).

m2. Inaccurate phrasing "integrating metabolite profiling and transcriptome analysis" in the

Discussion

Location: DISCUSSION, L164-165

The phrase "integrating metabolite profiling and transcriptome analysis" overstates the scope of

the metabolic measurements, because the study quantifies only bulk levels of major metabolite

classes (TPC, TFC, TAC, TPAC, TIC) rather than profiling individual metabolites.

To maintain accuracy and avoid implying a metabolite-level survey,

a more appropriate expression would be:

- "integrating metabolite measurements with transcriptome analysis"

- "combining transcriptome data with quantification of major metabolite classes"

This wording reflects the actual analytical scope of the study more precisely.

Response:

Thank you for your insightful comments. In the revised manuscript in Discussion section, we

have corrected the phrase now reads, “by integrating metabolite measurements with

transcriptome analysis”. (Line number - 384)

m3. Reference typo ("Skuba" → "Tsukuba")

Location: References, L809-810

The National Food Research Institute is located in Tsukuba, Japan.

Please correct the spelling.

Response:

We are sorry for our careless mistake. Now in the revised manuscript we have corrected the

spelling (Line number- 699).

m4. Statistical notation formatting

Location: RESULTS, L127 etc.

In scientific writing, statistical symbols such as P, q, and Q are conventionally italicized.

Please format all statistical values accordingly.

Response:

We are sorry for our careless mistake. All statistical symbols, including P, q, and Q, have been

reformatted to italic font throughout the Results section and also the entire manuscript.

m5. Figure 3 panel label mismatch

Location: Fig. 3 caption

Figure 3 uses (1) and (2) in the panels, but the caption uses (a) and (b).

Please align the caption with the figure.

Response:

We are sorry for our careless mistake. We have corrected the caption of Figure 3 (Revised

manuscript – Figure 2) to match the panel labels used in the figure with (a) and (b).

m6. Figure 4 caption order inconsistent with figure layout

Location: Fig. 4 caption

The figure shows F3H → ANS → LAR, but the caption lists ANS first.

Please correct to match the order in the figure.

Response:

Thank you for identifying this inconsistency. The caption of Figure 4 has been revised to reflect

the correct order of enzymes as presented in the figure (F3H-3 → ANS → LAR).

Summary

The manuscript presents valuable data and addresses an important biological question; However,

several critical issues need to be resolved to meet sound-science standards. In particular,

improvements are required in:

- methodological transparency, including extraction procedures, analytical conditions for HPLC

and spectrophotometric/radical-scavenging assays, and clear reporting of biological replicate

numbers for both metabolite assays and RT-qPCR.

- statistical reporting, such as specifying statistical tests, providing statistical P/q/Q values,

adding significance indicators in Figures 2 and 4, and appropriately referencing Table S1 when

discussing correlations.

- Accuracy and consistency of terminology, especially regarding the description of metabolite

measurements.

- organization and clarity of the Discussion, which is currently overly long and contains reviewstyle content not directly tied to the study's findings.

Addressing these points will substantially improve the rigor, reproducibility, and readability of

the manuscript.

Thank you for the comprehensive and constructive feedback. All specified issues have been

thoroughly addressed in the revised manuscript. Materials and methods section – with details

extraction protocols, HPLC conditions, biological replicates and statistical analysis. The

discussion part has been shortened on key study findings. We believe these changes and revised

manuscript will enhance rigor, reproducibility, and readability of the manuscript.
